# Ibrutinib suppresses LPS-induced neuroinflammatory responses in BV2 microglial cells and wild-type mice

**DOI:** 10.1186/s12974-018-1308-0

**Published:** 2018-09-19

**Authors:** Hye Yeon Nam, Jin Han Nam, Gwangho Yoon, Ju-Young Lee, Youngpyo Nam, Hye-Jin Kang, Hyun-Ji Cho, Jeongyeon Kim, Hyang-Sook Hoe

**Affiliations:** Department of Neural Development and Disease, Korea Brain Research Institute (KBRI), 61, Cheomdan-ro, Dong-gu, Daegu, 41068 South Korea

**Keywords:** LPS, Neuroinflammation, STAT3, AKT, Microglia

## Abstract

**Background:**

The FDA-approved small-molecule drug ibrutinib is an effective targeted therapy for patients with chronic lymphocytic leukemia (CLL). Ibrutinib inhibits Bruton’s tyrosine kinase (BTK), a kinase involved in B cell receptor signaling. However, the potential regulation of neuroinflammatory responses in the brain by ibrutinib has not been comprehensively examined.

**Methods:**

BV2 microglial cells were treated with ibrutinib (1 μM) or vehicle (1% DMSO), followed by lipopolysaccharide (LPS; 1 μg/ml) or PBS. RT-PCR, immunocytochemistry, and subcellular fractionation were performed to examine the effects of ibrutinib on neuroinflammatory responses. In addition, wild-type mice were sequentially injected with ibrutinib (10 mg/kg, i.p.) or vehicle (10% DMSO, i.p.), followed by LPS (10 mg/kg, i.p.) or PBS, and microglial and astrocyte activations were assessed using immunohistochemistry.

**Results:**

Ibrutinib significantly reduced LPS-induced increases in proinflammatory cytokine levels in BV2 microglial and primary microglial cells but not in primary astrocytes. Ibrutinib regulated TLR4 signaling to alter LPS-induced proinflammatory cytokine levels. In addition, ibrutinib significantly decreased LPS-induced increases in p-AKT and p-STAT3 levels, suggesting that ibrutinib attenuates LPS-induced neuroinflammatory responses by inhibiting AKT/STAT3 signaling pathways. Interestingly, ibrutinib also reduced LPS-induced BV2 microglial cell migration by inhibiting AKT signaling. Moreover, ibrutinib-injected wild-type mice exhibited significantly reduced microglial/astrocyte activation and COX-2 and IL-1β proinflammatory cytokine levels.

**Conclusions:**

Our data provide insights on the mechanisms of a potential therapeutic strategy for neuroinflammation-related diseases.

**Electronic supplementary material:**

The online version of this article (10.1186/s12974-018-1308-0) contains supplementary material, which is available to authorized users.

## Background

The human brain contains microglia, astrocytes, and neuronal cells. As resident macrophages in the central nervous system (CNS), microglia and astrocytes play vital roles in the innate immune response and serve as the frontline defense against exogenous toxic substances and proinflammatory reactions [1, 2]. In the normal brain, microglia play an important role in neuroprotection, and phagocytes remove cell debris and damaged neurons [3]. However, abnormally activated microglia and astrocytes significantly accelerate neuroinflammatory and neurotoxic responses by releasing various proinflammatory cytokines and mediators, including interleukin-1β (IL-1β), interleukin-6 (IL-6), tumor necrosis factor-α (TNF-α), cyclooxygenase-2 (COX-2), and nitric oxide synthase (iNOS) [4]. These neuroinflammatory responses are strongly correlated with neurodegenerative diseases such as Alzheimer’s disease (AD) and lead to synaptic degeneration, neuronal cell death, and cognitive dysfunction [5]. Therefore, regulation of the neuroinflammatory response represents a potential therapeutic strategy for neuroinflammation/neurodegeneration-related diseases.

Lipopolysaccharide (LPS) is a prominent cell wall component of gram-negative bacteria that is a strong stimulator of microglial activation [6]. LPS-induced microglial activation results in inflammatory responses that promote disease progression in models of neurodegeneration [7, 8]. LPS interacts with Toll-like receptors (TLRs) such as TLR4 on the surface of microglia [9]. This interaction activates TLR4 and downstream signaling pathways. Activated TLR4 signaling affects NF-κB and/or other transcription factors in the nucleus and triggers the release of proinflammatory cytokines [10]. Thus, modulating the LPS and TLR interaction and/or activation is a potential therapeutic strategy for preventing/treating neuroinflammation-related diseases.

Ibrutinib is an irreversible and selective small-molecule inhibitor of Bruton’s tyrosine kinase (BTK) [11] that can cross the blood-brain barrier [12]. BTK is a key regulator of B cell receptor functions and signaling and modulates cell survival and proliferation in various B cell malignancies. Anti-tumor activity of ibrutinib has been observed in vivo and in clinical studies [13]. According to several recent studies, ibrutinib has immunomodulatory action. For instance, treatment of mice with ibrutinib improved the anti-tumor immune response of infiltrating T cells [14]. Additionally, Kondo et al. showed that patients with chronic lymphocytic leukemia (CLL) who received ibrutinib exhibited significantly reduced STAT3 phosphorylation and IL-10 proinflammatory cytokine levels [15]. Ibrutinib is also a useful treatment for bone and autoimmune diseases, including rheumatoid arthritis [16]. As shown by Shinohara et al., oral administration of ibrutinib inhibits osteoclast resorption in the bone by targeting the integrin pathway [17]. However, researchers have not comprehensively investigated whether ibrutinib regulates neuroinflammatory responses in the brain.

In the present study, we examined the effects of ibrutinib on microglial and astrocytic proinflammatory responses and found that ibrutinib differentially regulates the neuroinflammatory responses in these cells. A decrease in TLR4/AKT/STAT3 signaling further suppressed proinflammatory cytokine levels as a downstream effect of ibrutinib. In addition, ibrutinib significantly suppressed LPS-induced BV2 microglial cell migration by modulating AKT signaling. Moreover, ibrutinib-injected wild-type mice exhibited significantly reduced microglial and astrocyte activation and decreased levels of the proinflammatory cytokines COX-2 and IL-1β. These data indicate that ibrutinib regulates LPS-stimulated neuroinflammatory responses in microglial cells and wild-type mice.

## Methods

### Cell lines and culture conditions

BV2 microglial cells (a generous gift from Dr. Kyung-Ho Suk) were maintained in high-glucose DMEM (Invitrogen, Carlsbad, CA, USA) with 5% fetal bovine serum (FBS, Invitrogen, Carlsbad, CA, USA) in a 5% CO_2_ incubator. Data from all in vitro experiments were analyzed in a semi-automated manner using ImageJ software, and the results were confirmed by an independent researcher who did not participate in the current experiments.

### Wild-type mice

All experiments were performed in accordance with approved animal protocols and guidelines established by the Korea Brain Research Institute (IACUC-2016-0013). C57BL6/N mice were purchased from Orient-Bio Company (Gyeonggi-do, Korea). Male C57BL6/N mice (8 weeks old, 25–30 g) were housed in a pathogen-free facility with 12 h of light and dark per day at an ambient temperature of 22 °C. Data were analyzed in a semi-automated manner using ImageJ software, and the results were confirmed by an independent researcher who did not participate in the current experiments.

### Immunohistochemistry

To determine whether pretreatment with ibrutinib alters LPS-induced neuroinflammation in vivo, wild-type mice were intraperitoneally (i.p.) administered ibrutinib (10 mg/kg) or vehicle (10% DMSO) daily for 3 days and subsequently injected with LPS (Sigma, *Escherichia coli*, 10 mg/kg, i.p.) or PBS. Three hours after the injection of LPS or PBS, the mice were perfused and fixed with 4% paraformaldehyde (PFA) solution, and the brain tissues were flash-frozen and sliced using a cryostat (35 μm thick). Each brain section was processed for immunohistochemical staining. The brain sections were rinsed with PBS and permeabilized with PBS containing 0.2% Triton X-100 and 0.5% BSA for 1 h at room temperature. The tissue sections were subsequently incubated with primary anti-Iba-1, anti-GFAP, anti-COX-2, or anti-IL-1β antibodies at 4 °C overnight. The next day, the tissue sections were washed with 0.5% BSA three times and incubated with a biotin-conjugated anti-rabbit secondary antibody (1:400, Vector Laboratories) for 1 h at room temperature. The sections were then rinsed with 0.5% BSA and incubated in an avidin-biotin complex solution (Vector Laboratories, Burlingame, CA) for 1 h at room temperature. After washing the sections three times with 0.1 M phosphate buffer (PB), the signal was detected by incubating the sections with 0.5 mg/ml 3,3′-diaminobenzidine (DAB, Sigma-Aldrich) in 0.1 M PB containing 0.003% H_2_O_2_. The sections were rinsed with 0.1 M PB and mounted on gelatin-coated slides, and images were captured under a bright-field microscope (Leica).

### Antibodies and inhibitors

The following primary antibodies were used for western blotting (WB) and immunocytochemistry (ICC): rat anti-mouse CD11b (1:400 for ICC, Abcam), rabbit anti-COX-2 (1:200 for ICC, Abcam), rabbit anti-IL-1β (1:200 for ICC, Abcam), rabbit anti-GFAP (1:500 for ICC, Wako, Japan), rabbit anti-Iba-1 (1:500 for ICC, Wako), goat anti-Iba-1 (1:500 for ICC, Wako), rabbit anti-AKT (1:1000 for WB, Santa Cruz Biotechnology), rabbit anti-p-AKT (Ser473) (1:1000 for WB, Cell Signaling Technology), rabbit anti-ERK (1:1000 for WB, Santa Cruz Biotechnology), rabbit anti-p-ERK (Thr42/44) (1:1000 for WB, Cell Signaling Technology), rabbit anti-STAT3 (1:1000 for WB, Cell Signaling Technology), rabbit anti-p-STAT3 (Ser727, 1:1000 for WB, 1:200 for ICC, Abcam), rabbit anti-JNK (1:1000 for WB, MyBioSource, San Diego, CA, USA), rabbit anti-p-JNK (Thr183/Tyr185, 1:1000 for WB, MyBioSource), rabbit anti-P38 (1:1000 for WB, Cell Signaling Technology), rabbit anti-p-P38 (1:1000 for WB, Cell Signaling Technology), rabbit anti-TLR4 (1:1000 for WB, Thermo Scientific, Waltham, MA, USA), and rabbit anti-TLR4 (1:1000 for WB, Novus Biologicals, Littleton, CO, USA). We used a TLR4 inhibitor (TAK-242, 500 nM, Calbiochem), AKT inhibitor (MK2206, 10 μM, Selleckchem), and STAT3 inhibitor (S3I-201, Sigma-Aldrich) in our experiments. LPS from *Escherichia coli* O111:B4 was purchased from Sigma-Aldrich (St. Louis, MO, USA).

### MTT assays

Cell viability was assessed using the 3-(4,5-dimethylthiazol-2-yl)-2,5-diphenyltetrazolium bromide (MTT) assay. BV2 microglial cells were seeded in 96-well plates and treated with various concentrations of ibrutinib (100 nM to 1 μM at lower doses and 1 μM to 50 μM at higher doses) for 24 h in the absence of FBS. The cells were then treated with 0.5 mg/ml MTT and incubated for 3 h at 37 °C in a 5% CO_2_ incubator. Absorbance was measured at 580 nm.

### Rat primary microglial and astrocyte cultures

Rat primary mixed glial cells were cultured from the cerebral cortices of 1-day-old Sprague-Dawley rats. Briefly, the cortices were triturated into single cells in high-glucose DMEM containing 10% FBS/penicillin-streptomycin solution (5000 units/ml penicillin, 5 mg/ml streptomycin, Corning, Mediatech Inc., Manassas, VA, USA) and plated into 75 T culture flasks (0.5 hemisphere/flask) for 2 weeks. To harvest rat primary microglial cells, the flask were shaken continuously at 120 rpm for 2 h to facilitate microglial detachment from the flask. The fluid medium was subsequently collected and centrifuged at 1500 rpm for 15 min, and the cell pellets were resuspended to plate 1 × 10^5^ cells per well. The remaining cells in the flask were harvested using 0.1% trypsin to obtain primary astrocytes. These primary astrocytes and primary microglial cells were cultured in 12-well plates (35 mm) pre-coated with poly-d-lysine (Sigma).

### Reverse transcription polymerase chain reaction

Total RNA was extracted using TRIzol (Invitrogen) according to the manufacturer’s instructions. Total RNA was reverse transcribed into cDNAs using a Superscript cDNA Premix Kit II with oligo (dT) primers (GeNetBio, Korea). RT-PCR was performed using Prime Taq Premix (GeNetBio, Korea). RT-PCR was performed using the following primers for BV2 microglial cells: IL-1β: forward (F)′, AGC TGG AGA GTG TGG ATC CC, and reverse (R) ′, CCT GTC TTG GCC GAG GAC TA; IL-6: F′, CCA CTT CAC AAG TCG GAG GC, and R′, GGA GAG CAT TGG AAA TTG GGG T; IL-18: F′, TTT CTG GAC TCC TGC CTG CT, and R′, ATC GCA GCC ATT GTT CCT GG; COX-2: F′, GCC AGC AAA GCC TAG AGC AA, and R′, GCC TTC TGC AGT CCA GGT TC; iNOS: F′, CCG GCA AAC CCA AGG TCT AC, and R′, GCA TTT CGC TGT CTC CCC AA; TNF-α: F′, CTA TGG CCC AGA CCC TCA CA, and R′, TCT TGA CGG CAG AGA GGA GG; and GAPDH: F′, CAG GAG CGA GAC CCC ACT AA, and R′, ATC ACG CCA CAG CTT TCC AG. For rat primary microglia and astrocytes, the following primers were used for RT-PCR: COX-2: F′, TCC AAC TCA AGT TCG ACC CA, and R′, TCC TCC GAA GGT GCT AGG TT; IL-1β: F′, AAA ATG CCT CGT GCT GTC TG, and R′, CAG AAT GTG CCA CGG TTT TC; IL-6: F′, TTG CCT TCT TGG GAC TGA TG, and R′, TGG AAG TTG GGG TAG GAA GG; iNOS: F′, ATC ATG GAC CAC CAC ACA GC, and R′, GGT GTT GAA GGC GTA GCT GA; TNF-α: F′, AGC ACA GAA AGC ATG ATC CG, and R′, CTC CCT CAG GGG TGT CCT TA; and GAPDH: F′, GTT ACC AGG GCT GCC TTC TC, and R′, GTG ATG GCA TGG ACT GTG GT. The RT-PCR products were separated by electrophoresis on 1.5% agarose gels containing eco dye (1:5000, Korea) and photographed. Images were analyzed using ImageJ (NIH) and Fusion software (Korea).

### Immunocytochemistry

BV2 microglial cells were fixed with 4% paraformaldehyde for 10 min, washed with PBS three times, and then incubated with anti-CD11b and anti-IL-1β or anti-CD11b and anti-COX-2 antibodies in GDB buffer (0.1% gelatin, 0.3% Triton X-100, 16 mM sodium phosphate, pH 7.4, and 450 mM NaCl) overnight at 4 °C. The next day, the cells were washed with PBS three times and incubated with the following secondary antibodies for 1 h at room temperature: Alexa Fluor 488-conjugated anti-mouse and Alexa Fluor 555-conjugated anti-rabbit (1:200, Molecular Probes, USA). The cells were mounted in DAPI-containing solution (Vector Laboratories, CA, USA), and images were captured from a single plane using a confocal microscope (Nikon, Japan) and analyzed using ImageJ software. Samples were analyzed in a blinded manner using 6–10 individual images.

### Enzyme-linked immunosorbent assay

To examine whether ibrutinib affects IL-1β levels, an enzyme-linked immunosorbent assay (ELISA) was performed. Briefly, BV2 microglial cells were treated with ibrutinib (500 nM) or vehicle (1% DMSO) for 30 min, treated with LPS (100 ng/ml) or PBS for 24 h. IL-1β ELISA was then performed using the conditioned medium. Mouse IL-1β ELISA kits (ELISA development reagents; R&D Systems, Minneapolis, MN) were used according to the manufacturer’s recommendations. Recombinant mouse IL-1β protein (R&D Systems) was used as a standard. The absorbance of the samples was measured at 450 nm using a microplate reader (BMG Labtech, Offenburg, Germany).

### Western blotting

To determine whether ibrutinib affects ERK, P38, JNK, and AKT signaling, BV2 microglial cells were treated with ibrutinib (1 μM) or vehicle (1% DMSO) for 1 h, followed by LPS (1 μg/ml) or PBS for 45 min. After the final incubation, the cells were lysed with RIPA buffer containing protease and phosphatase inhibitor tablets (Roche, USA). Western blot analyses were performed as previously described [18], and images were analyzed using Fusion software or ImageJ software.

### Wound-healing assay

Wound-healing assays were performed as previously described [18]. Briefly, BV2 microglial cells were seeded in 12-well plates and incubated until the cells reached 80–90% confluence. The cells were scratched with a cell scratcher (SPL, Korea) to create a wound. Images were captured at 0 h. Next, the cells were treated with ibrutinib (500 nM) or vehicle (1% DMSO) for 1 h, followed by LPS (100 ng/ml) or PBS for 23 h. Images were then captured.

### Cytosolic and nuclear fractionation

BV2 microglial cells were treated with ibrutinib (1 μΜ) or vehicle (1% DMSO) for 30 min, followed by treatment with LPS (1 μg/ml) or PBS for 5.5 h. The cells were then lysed in cytosol fractionation buffer (10 mM HEPES, pH 8.0, 1.5 mM MgCl_2_, 10 mM KCl, 0.5 mM DTT, 300 mM sucrose, 0.1% NP-40, and 0.5 mM PMSF). After 5 min, the cell lysates were centrifuged at 10,000 rpm for 1 min at 4 °C, and the supernatant was stored as the cytosolic fraction. The pellet was lysed in nuclear fractionation buffer (10 mM HEPES, pH 8.0, 20% glycerol, 100 mM KCl, 100 mM NaCl, 0.2 mM EDTA, 0.5 mM DTT, and 0.5 mM PMSF) on ice for 15 min. The sample was centrifuged at 10,000 rpm for 15 min at 4 °C. Western blot analyses were then performed with antibodies against p-STAT3 (Ser727), PCNA, and β-actin and analyzed using Fusion or ImageJ software.

### Cell surface biotinylation

To measure the effects of ibrutinib on cell-surface levels of TLR4, BV2 microglial cells were treated with ibrutinib (1 μM) or vehicle (1% DMSO) for 30 min, followed by treatment with LPS (1 μg/ml) or PBS for 5.5 h. Surface proteins were then labeled with Sulfo-NHS-SS-Biotin under gentle shaking at 4 °C for 30 min, followed by the addition of quenching solution. The surface-labeled cells were lysed in lysis buffer, disrupted by sonication on ice, incubated for 30 min, and clarified by centrifugation (10,000 rpm, 10 min). The lysate was then added to immobilized NeutroAvidin TM gel and incubated for 1 h, followed by washing three times with wash buffer and incubation for 1 h in SDS-PAGE sample buffer with DTT. Surface proteins were then analyzed by immunoblotting with an antibody recognizing the N-terminus of TLR4.

### Statistical analyses

All data were analyzed with GraphPad Prism 6 software using either unpaired two-tailed *t* tests with Welch’s correction for comparisons between two groups or one-way ANOVA for multiple comparisons. Post hoc analyses were performed with Tukey’s multiple comparison test with significance set at *p < 0.05, **p < 0.01, and ***p < 0.0001. Data are presented as the mean ± SEM.

## Results

### Ibrutinib does not exert toxic effects on BV2 microglial cells at concentrations up to 25 μM

**Fig. 1:**
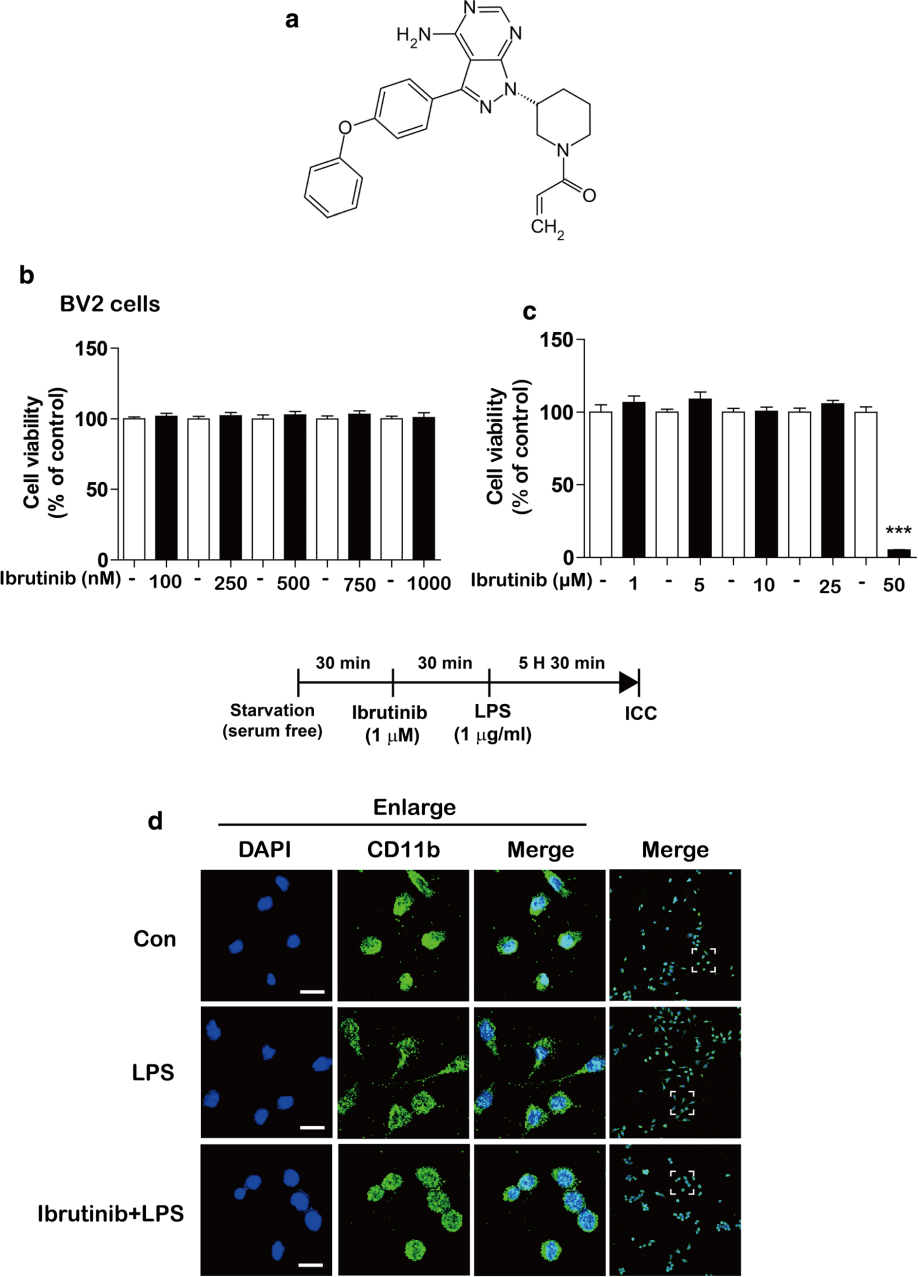
Ibrutinib did not exhibit toxicity toward BV2 microglial cells at concentrations up to 25 μM. **a** Structure of ibrutinib. **b** BV2 microglial cells were treated with vehicle (1% DMSO) or ibrutinib (100, 250, 500, 750, or 1000 nM) for 24 h, and MTT assays were performed (*n* = 16 replicates per dose). **c** BV2 microglial cells were treated with vehicle (1% DMSO) or ibrutinib (1, 5, 10, 25, or 50 μM) for 24 h, and cell viability was measured (*n* = 8 replicates per dose). **d** BV2 microglial cells were pretreated with ibrutinib (1 μM) or vehicle (1% DMSO) for 30 min, followed by treatment with LPS (1 μg/ml) or PBS for 5.5 h and immunostaining with an anti-CD11b antibody. Scale bar = 20 μm, ***p < 0.001

To test the effects of ibrutinib on neuroinflammation, we first examined whether ibrutinib is toxic toward BV2 microglial cells. BV2 microglial cells were treated with vehicle (1% DMSO) or ibrutinib (100, 250, 500, 750, or 1000 nM) for 24 h, and MTT assays were conducted. Ibrutinib did not exhibit toxicity at lower doses (Fig. 1a, b). We also examined the effects of higher doses of ibrutinib on cell viability. For this purpose, BV2 microglial cells were treated with vehicle (1% DMSO) or ibrutinib (1, 5, 10, 25, or 50 μM) for 24 h, and MTT assays were conducted. We found that ibrutinib did not induce BV2 microglial cell toxicity at concentrations up to 25 μM (Fig. 1c).

To determine whether ibrutinib alters the LPS-induced morphology of BV2 microglial cells, cells were pretreated with ibrutinib (1 μM) or vehicle (1% DMSO) for 30 min, followed by treatment with LPS (1 μg/ml) or PBS for 5.5 h. The cells were then fixed in 4% paraformaldehyde and immunostained with an anti-CD11b antibody. In contrast to vehicle-treated cells, LPS-treated BV2 microglial cells displayed long thin processes extending from the cell body (Fig. 1d, middle panel). Pretreatment with ibrutinib followed by LPS treatment reduced the number of long thin processes extending from the cell body, and the morphology of these cells resembled that of vehicle-treated cells (Fig. 1d, lower panel).

### Ibrutinib significantly decreases LPS-induced proinflammatory cytokine levels

**Fig. 2:**
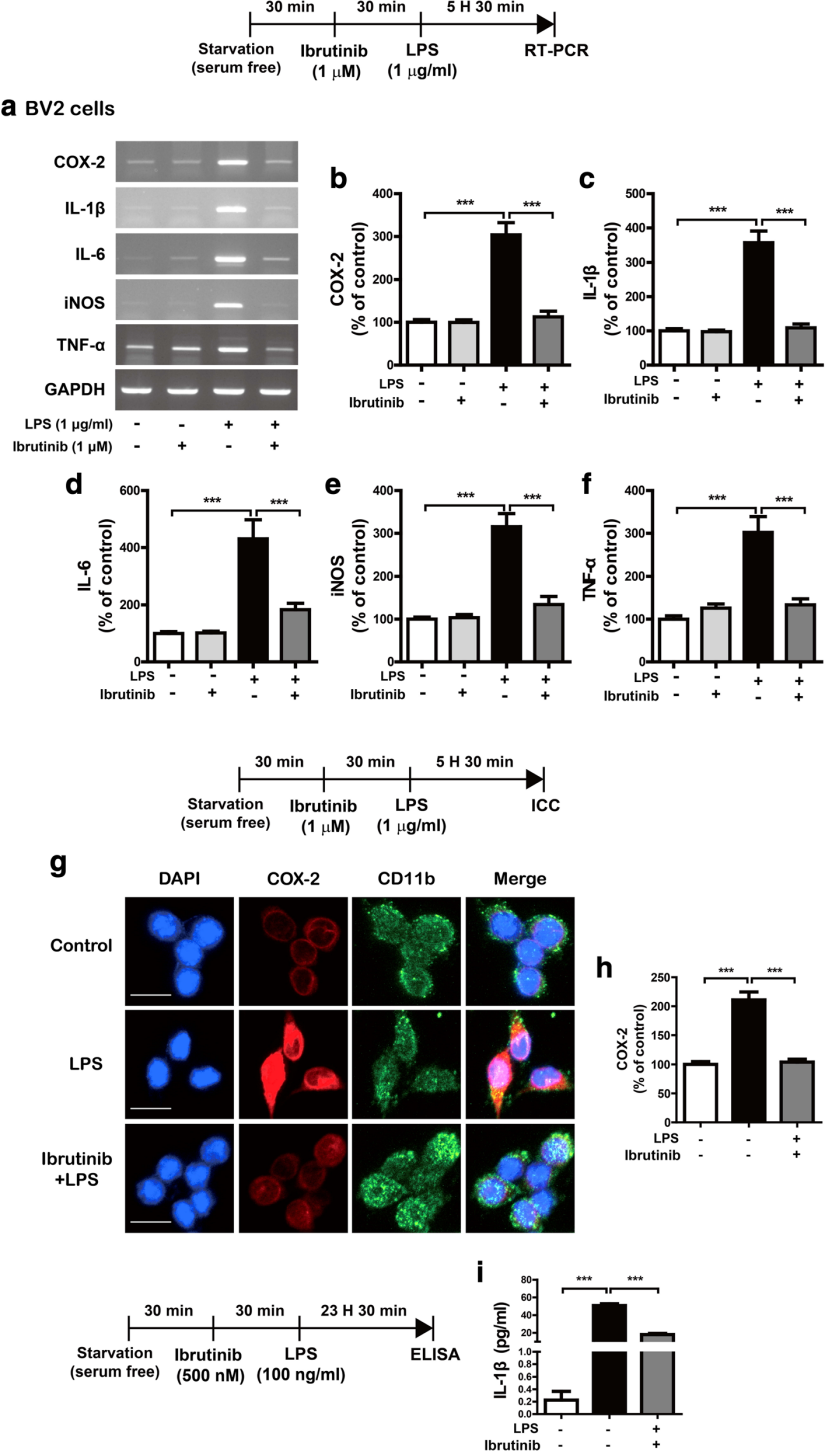
Ibrutinib significantly decreased LPS-induced proinflammatory cytokine levels in BV2 microglial cells. **a** BV2 microglial cells were treated with vehicle (1% DMSO) or ibrutinib (1 μM) for 30 min, followed by treatment with PBS or LPS (1 μg/ml) for 5.5 h, and RT-PCR was performed. **b**–**f** Quantification of the data shown in **a** (COX-2, IL-1β, IL-6, iNOS, and TNF-α: con, *n* = 15; ibrutinib, *n* = 15; LPS, *n* = 15; ibrutinib+LPS, *n* = 15). **g** BV2 microglial cells were treated with vehicle (1% DMSO) or ibrutinib (1 μM) for 30 min, followed by PBS or LPS (1 μg/ml) for 5.5 h, and immunocytochemistry was conducted with anti-CD11b and anti-COX-2 antibodies. **h** Quantification of the data shown in **g** (COX-2: con, *n* = 77; LPS, *n* = 73; ibrutinib+LPS, *n* = 112). **i** BV2 microglial cells were treated with vehicle (1% DMSO) or ibrutinib (500 nM) for 30 min, treated with LPS (100 ng/ml) or PBS for 24 h, and measured IL-1β levels using IL-1β ELISA (con, *n* = 10; LPS, *n* = 10; LPS+ibrutinib, *n* = 10). Scale bar = 20 μm, ***p < 0.001

To investigate the potential regulatory effects of ibrutinib on proinflammatory responses, BV2 microglial cells were treated with vehicle (1% DMSO) or ibrutinib (1 μM) for 30 min, followed by LPS (1 μg/ml) or PBS for 5.5 h. Total RNA was isolated, and proinflammatory cytokine levels were measured using RT-PCR. Ibrutinib alone did not alter any proinflammatory cytokine levels compared with vehicle treatment without LPS (Fig. 2a–f). However, ibrutinib significantly reduced the mRNA levels of the LPS-induced proinflammatory cytokines COX-2, IL-6, IL-1β, iNOS, and TNF-α (Fig. 2a–f). As an alternative approach, we performed immunocytochemistry. For this experiment, BV2 microglial cells were treated with vehicle (1% DMSO) or ibrutinib (1 μM) for 30 min, followed by LPS (1 μg/ml) or PBS for 5.5 h. Then, immunostaining was performed with anti-CD11b and anti-COX-2 antibodies. Ibrutinib significantly decreased LPS-induced COX-2 levels in BV2 microglial cells (Fig. 2g, h). To further confirm these findings, we conducted an IL-1β ELISA assay. To test this, BV2 microglial cells were pretreated with ibrutinib (500 nM) or vehicle (1% DMSO) for 30 min, treated with LPS (100 ng/ml) or PBS for 24 h, and the IL-1β ELISA assay was performed. Consistent with the findings above, ibrutinib significantly reduced LPS-induced IL-1β levels compared with LPS treatment (Fig. 2i). These results indicate that pretreatment with ibrutinib regulates LPS-mediated increases in proinflammatory responses in BV2 microglial cells.

We then investigated whether post-treatment with ibrutinib alters LPS-stimulated proinflammatory responses. BV2 microglial cells were treated with LPS (1 μg/ml) or PBS for 30 min, followed by vehicle (1% DMSO) or ibrutinib (1 μM) for 5.5 h. Then, total RNA was isolated, and proinflammatory cytokine levels were measured by RT-PCR. Again, treatment with ibrutinib itself did not reduce the levels of any proinflammatory cytokines compared with vehicle treatment without LPS (Additional file 1: Figure S1a–f). However, post-treatment with ibrutinib significantly decreased the LPS-induced mRNA levels of COX-2, IL-6, and iNOS levels but not those of other proinflammatory cytokines (Additional file 1: Figure S1a–f). Thus, these data suggest that pretreatment (as a preventive measure) or post-treatment (as a curative treatment) with ibrutinib differentially regulates LPS-stimulated proinflammatory responses in BV2 microglial cells.

### Ibrutinib suppresses LPS-stimulated increases in proinflammatory cytokine levels in primary microglial cells

**Fig. 3:**
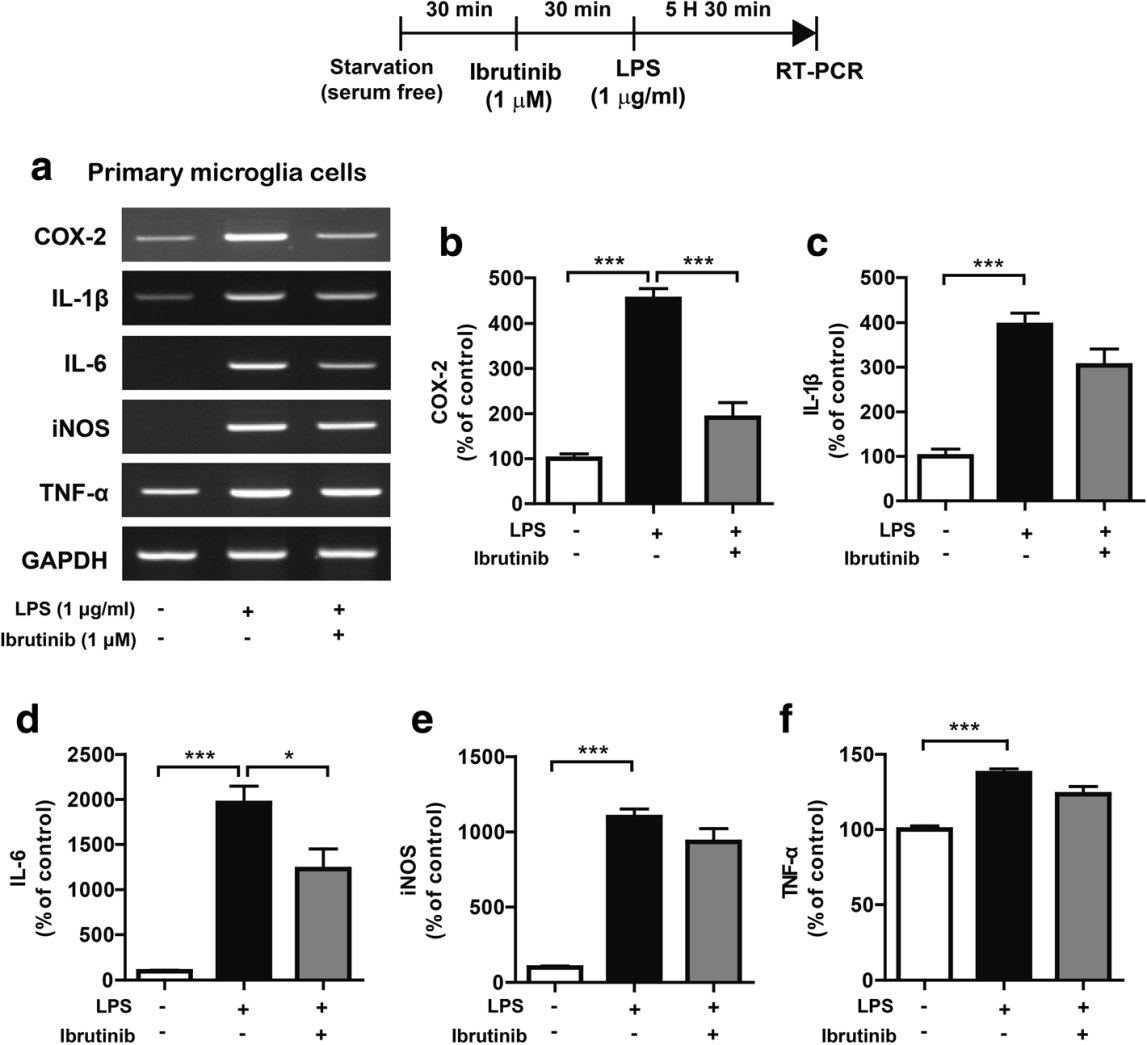
Ibrutinib significantly reduced the LPS-induced increases in the mRNA levels of the proinflammatory cytokines COX-2 and IL-6 in primary microglial cells. **a** Primary microglial cells were treated with vehicle (1% DMSO) or ibrutinib (1 μM) for 30 min, followed by PBS or LPS (1 μg/ml) for 5.5 h, and RT-PCR was conducted. **b**–**f** Quantification of the data shown in **a** (COX-2, IL-1β, IL-6, iNOS, and TNF-α: con, *n* = 4; LPS, *n* = 4; ibrutinib+LPS, *n* = 4). *p < 0.05, ***p < 0.001

To determine whether ibrutinib regulates LPS-induced proinflammatory responses in primary glial cells, primary microglial cells were treated with vehicle (1% DMSO) or ibrutinib (1 μM) for 30 min, followed by LPS (1 μg/ml) or PBS for 5.5 h. Proinflammatory cytokine levels were then measured by RT-PCR. LPS treatment significantly upregulated the levels of proinflammatory cytokines (Fig. 3a–f). By contrast, ibrutinib significantly downregulated the LPS-mediated increases in the levels of the mRNAs encoding the proinflammatory cytokines COX-2 and IL-6 in primary microglial cells (Fig. 3a–f).

We then examined whether ibrutinib also affects LPS-stimulated proinflammatory cytokine levels in primary astrocytes. Primary astrocytes were treated with vehicle (1% DMSO) or ibrutinib (1 μM) for 30 min, followed by LPS (1 μg/ml) or PBS for 5.5 h, and then proinflammatory cytokine levels were measured by RT-PCR. Surprisingly, ibrutinib did not reduce the levels of any proinflammatory cytokines in LPS-induced primary astrocytes (Additional file 1: Figure S2a–f). These data suggest that ibrutinib selectively affects proinflammatory responses depending on the cell type.

### Ibrutinib regulates TLR4 to alter LPS-induced proinflammatory cytokine levels

**Fig. 4:**
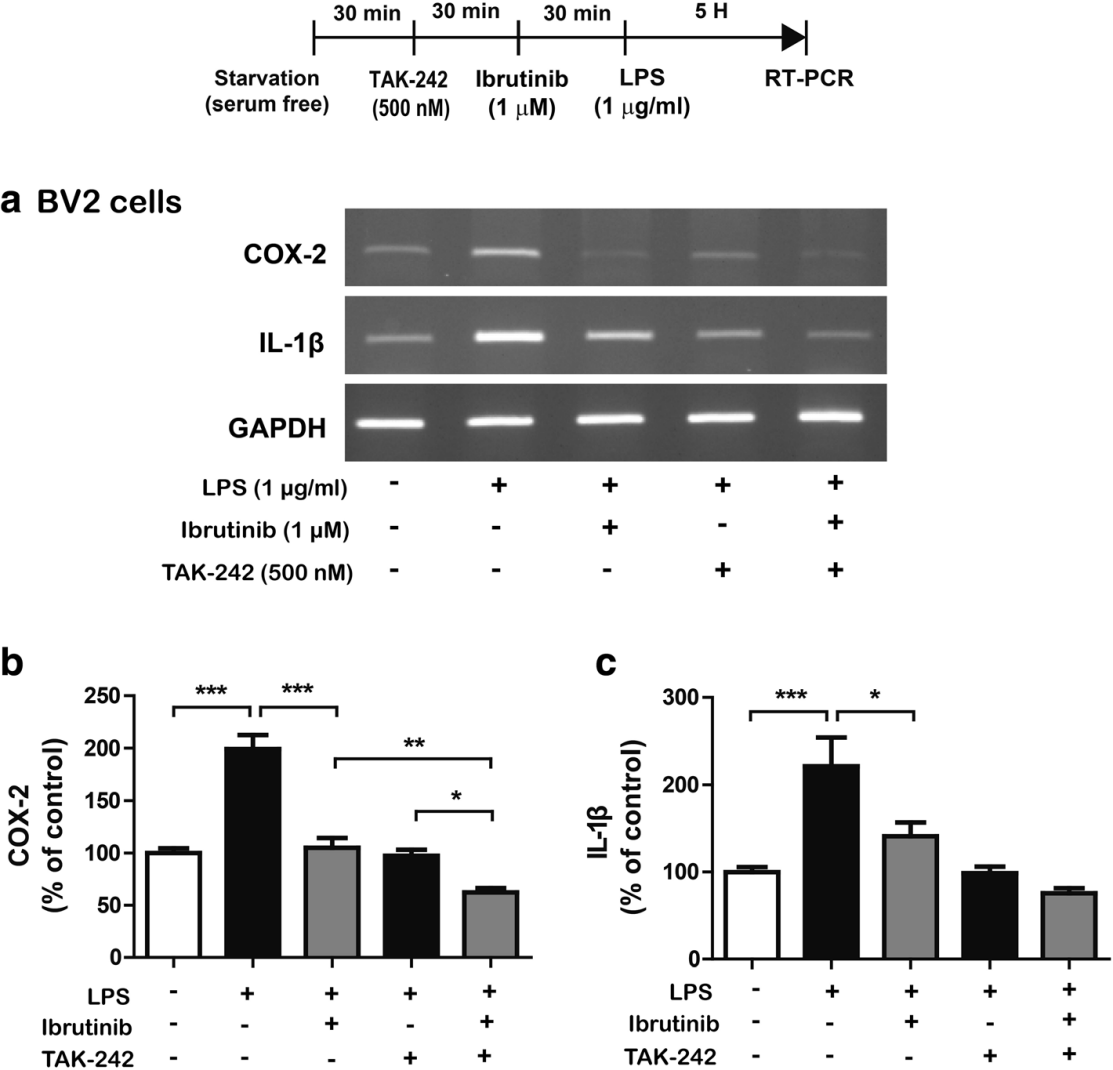
Ibrutinib modulates TLR4 to alter LPS-induced proinflammatory cytokine levels. **a** BV2 microglial cells were pretreated with TAK-242 (a TLR4 receptor inhibitor, 500 nM) or vehicle (1% DMSO) for 30 min, followed by treatment with ibrutinib (1 μM) or vehicle (1% DMSO) for 30 min, and finally LPS (1 μg/ml) or PBS for 5 h. The mRNA levels of IL-1β and COX-2 were measured by RT-PCR. **b**, **c** Quantification of the data shown in **a** (COX-2: con, *n* = 14; LPS, *n* = 14; ibrutinib+LPS, *n* = 14; TAK-242+LPS, *n* = 14; TAK-242+ibrutinib+LPS, *n* = 14; and IL-1β: con, *n* = 8; LPS, *n* = 8; ibrutinib+LPS, *n* = 8; TAK-242 + LPS, *n* = 8; TAK-242+ibrutinib+LPS, *n* = 8)

LPS interacts with TLR4 on the surface of microglial cells to increase immune responses [19]. Thus, we hypothesized that ibrutinib may inhibit the LPS and TLR4 interaction on the cell surface and/or TLR4 activation to regulate neuroinflammatory responses. To test this hypothesis, BV2 microglial cells were pretreated with TAK-242 (TLR inhibitor, 500 nM) or vehicle (1% DMSO) for 30 min, followed by treatment with ibrutinib (1 μM) or vehicle (1% DMSO) for 30 min and subsequent treatment with LPS (1 μg/ml) or PBS for 5 h. Total RNA was isolated, and IL-1β and COX-2 mRNA levels were measured by RT-PCR. Consistent with our findings described above, ibrutinib significantly decreased LPS-induced COX-2 and IL-1β mRNA levels (Fig. 4a–c). In addition, treatment with TAK-242, ibrutinib, and LPS further decreased LPS-induced COX-2 mRNA levels compared with treatment with LPS and TAK-242 or ibrutinib and LPS (Fig. 4a–c). However, treatment with TAK-242, ibrutinib, and LPS did not significantly reduce LPS-induced IL-1β mRNA levels compared with treatment with LPS and TAK-242 or ibrutinib and LPS (Fig. 4a–c).

We then examined whether ibrutinib modulates cell-surface levels of TLR4. BV2 microglial cells were treated with ibrutinib (1 μM) or vehicle (1% DMSO) for 30 min, followed by treatment with LPS (1 μg/ml) or PBS for 5.5 h, and cell-surface biotinylation was performed. We found that LPS treatment significantly increased cell-surface levels of TLR4 (Additional file 1: Figure S3a, b). However, ibrutinib reduced LPS-induced cell-surface levels of TLR4 compared with LPS treatment without changing total TLR4 levels (Additional file 1: Figure S3a, b, ibrutinib+LPS vs LPS, *p* = 0.1, by 68.7%). These data suggest that ibrutinib may regulate cell-surface levels of TLR4 and potentially inhibit interactions between TLR4 and LPS on the cell surface to alter LPS-stimulated proinflammatory responses.

### Ibrutinib alters AKT signaling to modulate LPS-induced proinflammatory responses

According to several recent studies, MAP kinase signaling (e.g., ERK, JNK, p-P38, AKT) plays important roles in modulating proinflammatory cytokine levels in glial cells [20, 21]. Thus, we examined whether ibrutinib down- or upregulates LPS-induced MAP kinase signaling. BV2 microglial cells were pretreated with ibrutinib (1 μM) or vehicle (1% DMSO) for 1 h, followed by treatment with LPS (1 μg/ml) or PBS for 45 min, and western blotting was conducted with anti-p-ERK/ERK, anti-p-JNK/JNK, or anti-p-P38/P38 antibodies. Unexpectedly, ibrutinib did not reduce the LPS-mediated increases in p-ERK levels in BV2 microglial cells (Additional file 1: Figure S4a–c). Again, we observed that ibrutinib did not suppress LPS-induced p-JNK (Additional file 1: Figure S4d–f) and p-P38 (Additional file 1: Figure S4g–i) levels in BV2 microglial cells.

**Fig. 5:**
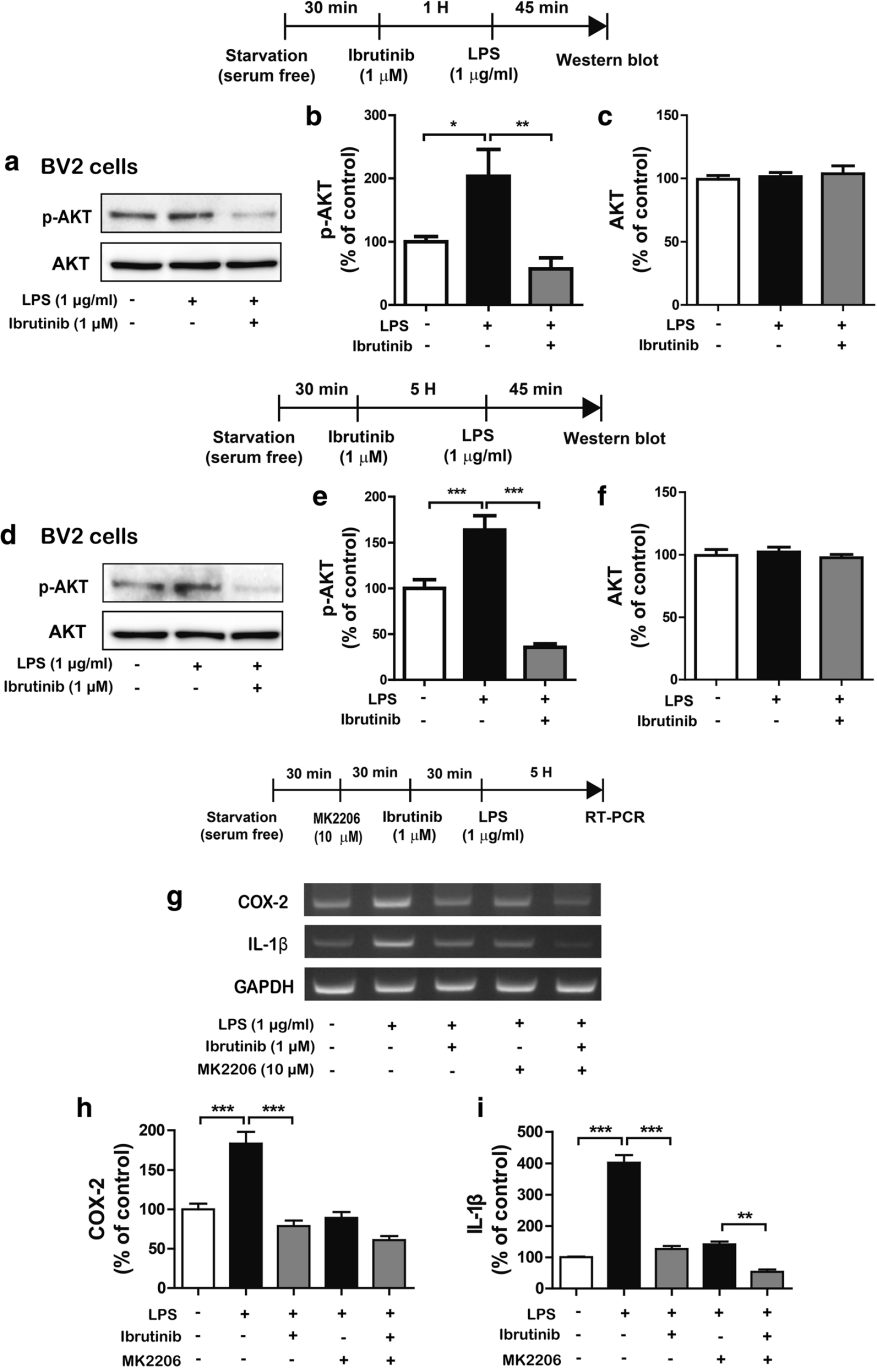
Ibrutinib significantly decreased LPS-induced AKT phosphorylation. **a** BV2 microglial cells were treated with vehicle (1% DMSO) or ibrutinib (1 μM) for 1 h, followed by treatment with PBS or LPS (1 μg/ml) for 45 min and western blotting with anti-p-AKT and anti-AKT antibodies. **b**, **c** Quantification of the data shown in **a** (p-AKT and AKT: con, *n* = 9; LPS, *n* = 9; ibrutinib+LPS, *n* = 9). **d** BV2 microglial cells were treated with vehicle (1% DMSO) or ibrutinib (1 μM) for 5 h, followed by treatment with PBS or LPS (1 μg/ml) for 45 min and western blotting with anti-p-AKT and anti-AKT antibodies. **e**, **f** Quantification of the data shown in **d (**p-AKT: con, *n* = 10; LPS, *n* = 10; ibrutinib+LPS, *n* = 10; and AKT: con, *n* = 9; LPS, *n* = 9; ibrutinib+LPS, *n* = 9). **g** BV2 microglial cells were treated with MK2206 (AKT inhibitor, 10 μM) or vehicle (1% DMSO) for 30 min, vehicle (1% DMSO) or ibrutinib (1 μM) for 30 min, and finally treated with PBS or LPS (1 μg/ml) for 5 h, and RT-PCR was performed. **h**, **i** Quantification of the data shown in **g** (COX-2: con, *n* = 13; LPS, *n* = 13; ibrutinib+LPS, *n* = 13; MK2206+LPS, *n* = 13; MK2206+ibrutinib+LPS, *n* = 13; and IL-1β: con, *n* = 4; LPS, *n* = 4; ibrutinib+LPS, *n* = 4; MK2206+LPS, *n* = 4; MK2206+ibrutinib+LPS, *n* = 4). *p < 0.05, **p < 0.01, ***p < 0.001

We then examined whether ibrutinib alters LPS-induced p-AKT signaling. To test this, BV2 microglial cells were treated with ibrutinib (1 μM) or vehicle (1% DMSO) for 1 h, followed by treatment with LPS (1 μg/ml) or PBS for 45 min, and western blotting was conducted with anti-p-AKT and anti-AKT antibodies (Fig. 5a–c). We found that ibrutinib significantly reduced the LPS-induced increases in p-AKT levels in BV2 microglial cells (Fig. 5a–c).

Next, we investigated the effects of ibrutinib on LPS-induced p-AKT levels in a longer treatment. BV2 microglial cells were pretreated with ibrutinib (1 μM) or vehicle (1% DMSO) for 5 h, followed by treatment with LPS (1 μg/ml) or PBS for 45 min, and western blotting was performed with anti-p-AKT and anti-AKT antibodies. Longer treatment with ibrutinib significantly reduced the LPS-mediated increases in p-AKT levels compared with LPS treatment (Fig. 5d–f). In addition, we measured whether ibrutinib itself affects AKT phosphorylation in the absence of LPS. For this experiment, BV2 microglial cells were treated with ibrutinib (1 μM) or vehicle (1% DMSO) for 6 h, and western blotting was performed with anti-p-AKT and anti-AKT antibodies. Unexpectedly, ibrutinib alone significantly reduced p-AKT levels in BV2 microglial cells, but the total AKT levels were unchanged (Additional file 1: Figure S5a–c).

To test whether ibrutinib modulates AKT signaling to alter LPS-mediated proinflammatory responses, BV2 microglial cells were pretreated with the MK2206 (a AKT inhibitor, 10 μM) or vehicle (1% DMSO) for 30 min, treated with ibrutinib (1 μM) or vehicle (1% DMSO) for 30 min, treated with LPS (1 μg/ml) or PBS for 5 h, and mRNA levels of COX-2 and IL-1β were measured by RT-PCR. Treatment with MK2206, ibrutinib, and LPS significantly suppressed the mRNA levels of IL-1β compared with treatment with ibrutinib and LPS or MK2206 and LPS (Fig. 5g–i). By contrast, treatment with MK2206, ibrutinib, and LPS did not significantly reduce the mRNA levels of COX-2 compared with treatment with ibrutinib and LPS or MK2206 and LPS (Fig. 5g–i). These data indicate that the effects of ibrutinib on proinflammatory responses in LPS-induced BV2 microglial cells partially depend on AKT signaling.

### Ibrutinib significantly decreases LPS-stimulated p-STAT3 levels in the nucleus

**Fig. 6:**
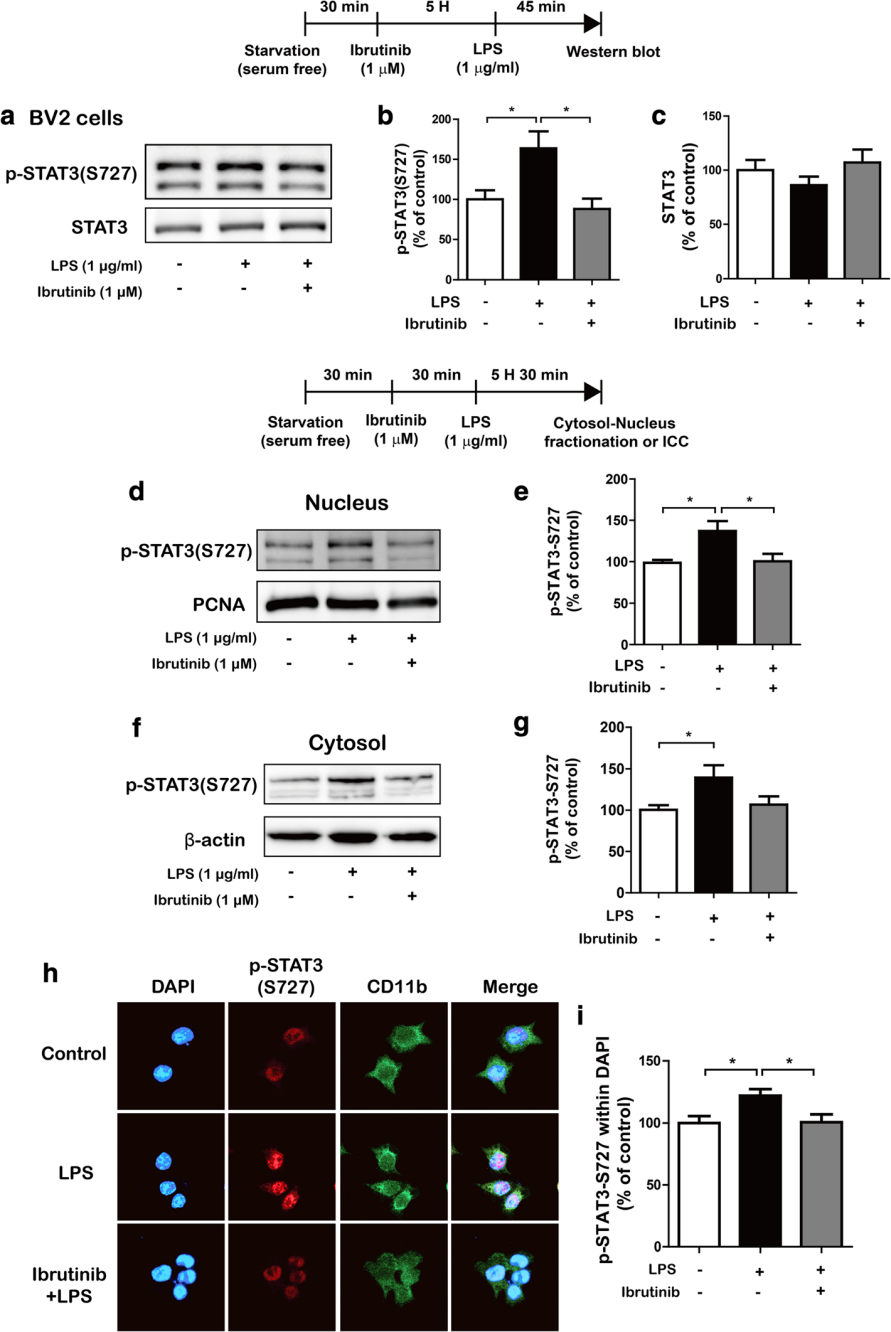
Ibrutinib significantly reduced LPS-induced STAT3 phosphorylation. **a**–**c** BV2 microglial cells were treated with vehicle (1% DMSO) or ibrutinib (1 μM) for 5 h, followed by treatment with PBS or LPS (1 μg/ml) for 45 min and western blotting with anti-p-STAT3 (Ser727) and anti-STAT3 antibodies. **b**, **c** Quantification of the data shown in **a** (p-STAT3 (Ser727) and STAT3: con, *n* = 6, LPS, *n* = 6 ibrutinib+LPS, *n* = 6). **d** BV2 microglial cells were treated with vehicle (1% DMSO) or ibrutinib (1 μM) for 30 min, followed by treatment with PBS or LPS (1 μg/ml) for 5.5 h and western blotting of the nuclear fraction with anti-p-STAT3 (Ser727) and anti-PCNA (as a nuclear marker) antibodies. **e** Quantification of the data shown in **d** (con, *n* = 10; LPS, *n* = 10; ibrutinib+LPS, *n* = 10). **f** BV2 microglial cells were treated with vehicle (1% DMSO) or ibrutinib (1 μM) for 30 min, followed by treatment with PBS or LPS (1 μg/ml) for 5.5 h and western blotting of the cytosolic fraction with anti-p-STAT3 (Ser 727) and anti-β-actin (as a cytosolic marker) antibodies. **g** Quantification of the data shown in **f** (con, *n* = 10; LPS, *n* = 10; ibrutinib+LPS, *n* = 10). **h** BV2 microglial cells were sequentially treated with vehicle (1% DMSO) or ibrutinib (1 μM) for 30 min and PBS or LPS (1 μg/ml) for 5.5 h, followed by immunocytochemistry with anti-p-STAT3 (Ser 727) and anti-CD11b antibodies. **i** Quantification of the data shown in **h** (p-STAT3 (Ser 727): con, *n* = 64; LPS, *n* = 77; ibrutinib+LPS, *n* = 90). *p < 0.05

The transcription factor signal transducer and activator of transcription 3 (STAT3) is a key signaling molecule that regulates proinflammatory cytokine levels following treatment with LPS [22]. We examined whether ibrutinib modulates LPS-induced p-STAT3 (Ser727) levels. BV2 microglial cells were treated with ibrutinib (1 μM) or vehicle (1% DMSO) for 5 h, followed by treatment with LPS (1 μg/ml) or PBS for 45 min, and western blotting was performed with anti-p-STAT3 (Ser727) and anti-STAT3 antibodies. We found that ibrutinib reduced LPS-induced p-STAT3 (Ser727) levels in BV2 microglial cells (Fig. 6a–c). We then tested whether ibrutinib alone alters p-STAT3 (Ser727) levels in BV2 microglial cells. BV2 microglial cells were treated with ibrutinib (1 μM) or vehicle (1% DMSO) for 6 h, and western blotting was conducted with anti-p-STAT3 (Ser727) and anti-STAT3 antibodies. Interestingly, ibrutinib itself downregulated p-STAT3 (Ser727) levels compared with vehicle treatment in the absence of LPS (Additional file 1: Figure S5).

Next, we examined whether ibrutinib modulates LPS-induced cytosolic and nuclear p-STAT3 (Ser727) levels. BV2 microglial cells were pretreated with ibrutinib (1 μM) or vehicle (1% DMSO) for 30 min, followed by treatment with LPS (1 μg/ml) or PBS for 5.5 h, and subcellular fractionation was conducted. Compared with the vehicle treatment, LPS treatment significantly increased p-STAT3 (Ser727) levels in the nucleus (Fig. 6d, e) and cytosol (Fig. 6f, g). In addition, ibrutinib significantly reduced the LPS-stimulated increases in nuclear p-STAT3 (Ser727) levels (Fig. 6d, e), with a trend toward decreased cytosolic p-STAT3 (Ser727) levels (Fig. 6f, g). To verify these findings, we performed immunocytochemistry with anti-p-STAT3 (Ser727) and anti-CD11b antibodies and found that ibrutinib significantly downregulated LPS-induced nuclear p-STAT3 (Ser727) levels in BV2 microglial cells (Fig. 6h, i). These data suggest that ibrutinib regulate p-STAT3 (Ser727) levels in the cytosol and nucleus to modify LPS-induced proinflammatory responses.

To investigate whether ibrutinib regulates AKT signaling to alter LPS-induced p-STAT3 (Ser727) levels in the nucleus, BV2 microglial cells were pretreated with the MK2206 (AKT inhibitor, 10 μM) or vehicle (1% DMSO) for 30 min, followed by treatment with ibrutinib (1 μM) or vehicle (1% DMSO) for 30 min and subsequent treatment with LPS (1 μg/ml) or PBS for 5 h. Immunocytochemistry was performed with anti-CD11b and anti-p-STAT3 (Ser727) antibodies. Consistent with our findings above, ibrutinib significantly decreased LPS-induced nuclear p-STAT3 (Ser727) levels compared with LPS treatment (Additional file 1: Figure S6a, b). Treatment with MK2206 and LPS significantly reduced LPS-induced nuclear p-STAT3 (Ser727) levels compared with LPS treatment. In addition, treatment with MK2206, ibrutinib, and LPS significantly suppressed LPS-induced nuclear p-STAT3 (Ser727) levels compared with treatment with MK2206 and LPS (Additional file 1: Figure S6a, b). These data suggest that ibrutinib may affect LPS-induced nuclear p-STAT3 (Ser727) levels in an AKT-independent manner.

### Ibrutinib significantly reduces LPS-induced BV2 microglial cell migration via AKT signaling

**Fig. 7:**
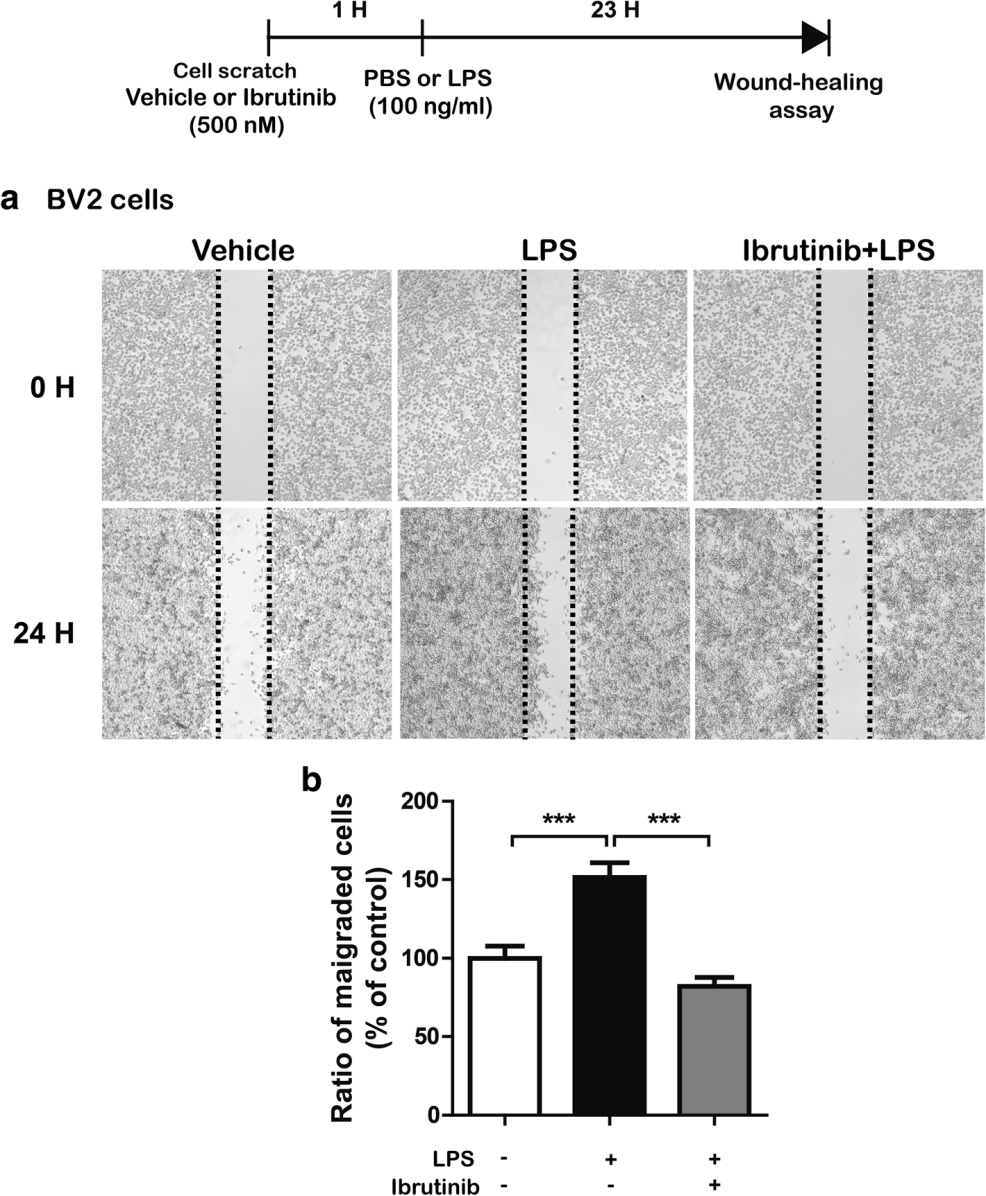
Ibrutinib significantly reduced LPS-induced BV2 microglial cell migration. **a** Monolayers of BV2 microglial cells were scratched with a fine tip, pretreated with vehicle (1% DMSO) or ibrutinib (500 nM) for 1 h, and then treated with PBS or LPS (100 ng/ml) for 23 h. Images of the wound gap were acquired at 0 h (i.e., immediately after scratching) and 24 h. **b** Quantification of the data shown in **a** (con, *n* = 22; LPS, *n* = 22; ibrutinib+LPS, *n* = 22). ***p < 0.001

Microglial cell movement is associated with proinflammatory responses [23]. Therefore, we examined whether ibrutinib regulates LPS-induced BV2 microglial cell migration. We performed wound-healing assays by pretreating cells with ibrutinib (500 nM) or vehicle (1% DMSO) for 1 h, followed by LPS (100 ng/ml) or PBS for 23 h. LPS treatment significantly increased BV2 microglial cell migration compared with vehicle treatment (Fig. 7a, b). In addition, ibrutinib significantly decreased LPS-stimulated BV2 microglial cell migration compared with LPS treatment (Fig. 7a, b). We then investigated whether ibrutinib itself regulates BV2 microglial cell migration. BV2 microglial cells were scratched using a fine pipette tip and immediately imaged (0 h). The cells were then treated with ibrutinib (500 nM) or vehicle (1% DMSO) for 24 h, followed by imaging of the wound gap. We found that ibrutinib alone did not reduce BV2 microglial cell migration compared with vehicle treatment in the absence of LPS (Additional file 1: Figure S7). These data suggest that ibrutinib regulates LPS-induced BV2 microglial cell migration.

**Fig. 8:**
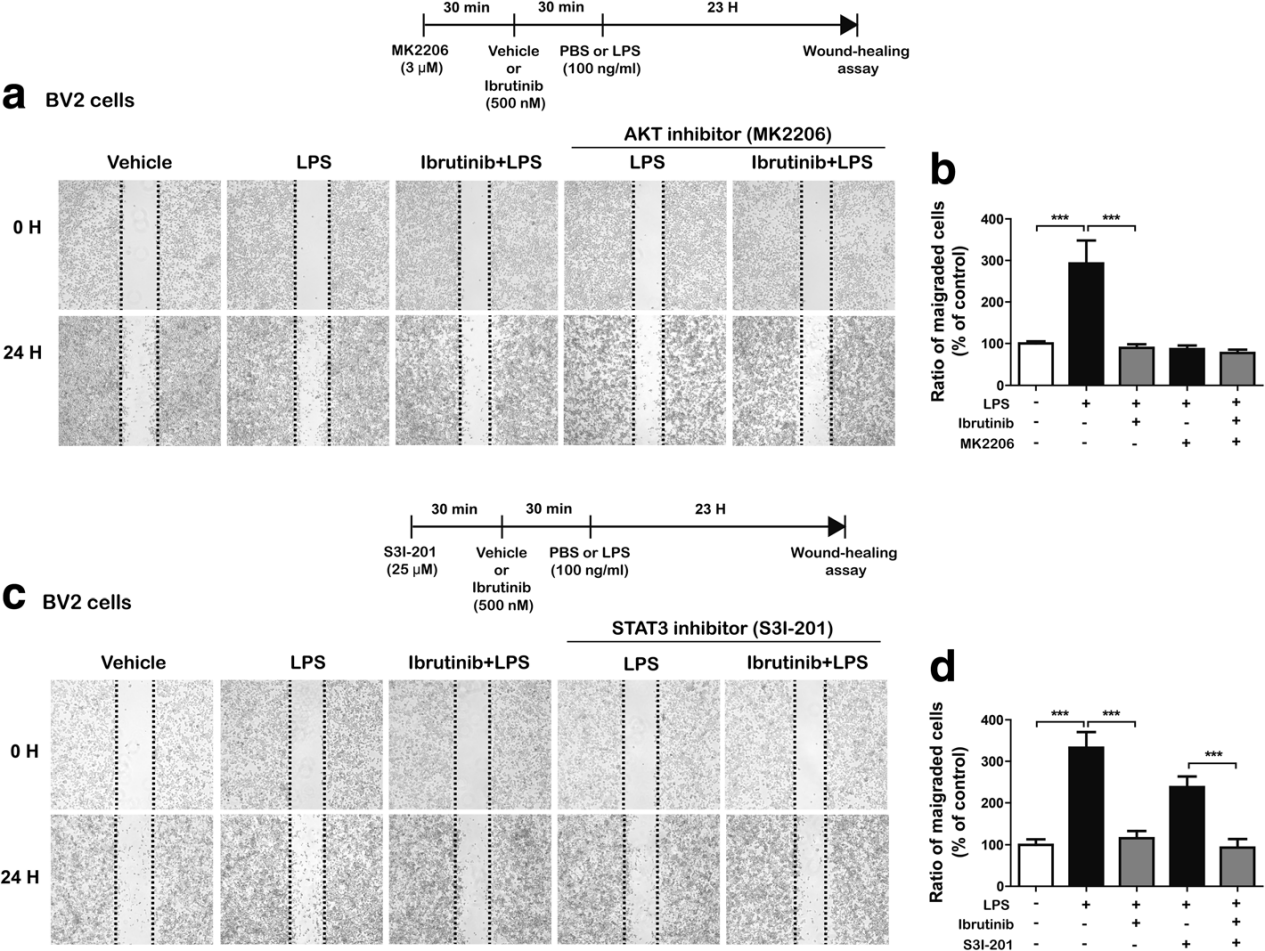
Ibrutinib alters LPS-induced BV2 microglial cell migration through AKT signaling, but not STAT3 signaling. **a** BV2 microglial cell monolayers were scratched with a fine tip, pretreated with MK2206 (AKT inhibitor, 3 μM) or vehicle (1% DMSO) for 30 min, treated with ibrutinib (500 nM) or vehicle (1% DMSO) for 30 min, and then treated with LPS (100 ng/ml) or PBS for 23 h. Images of the wound gap were acquired at 0 h (i.e., immediately after scratching) and after 24 h. **b** Quantification of data from **a** (con, *n* = 41; LPS, *n* = 42; ibrutinib+LPS, *n* = 41; MK2206+LPS, *n* = 38; ibrutinib+MK2206+LPS, *n* = 41). **c** BV2 microglial cell monolayers were scratched with a fine tip, pretreated with S31-201 (STAT3 inhibitor, 25 μM) or vehicle (1% DMSO) for 30 min, treated with ibrutinib (500 nM) or vehicle (1% DMSO) for 30 min, and then treated with LPS (100 ng/ml) or PBS for 23 h. Images of the wound gap were acquired at 0 h (i.e., immediately after scratching) and after 24 h. **d** Quantification of data from **c** (con, *n* = 24; LPS, *n* = 25; ibrutinib+LPS, *n* = 26; S31-201+LPS, *n* = 25; ibrutinib+S31-201+LPS, *n* = 24)

We then further investigated whether ibrutinib alters LPS-induced BV2 microglial cell migration via AKT signaling. BV2 microglial cells were scratched using a fine pipette tip and immediately imaged (0 h), followed by treatment with MK2206 (an AKT inhibitor, 3 μM) or vehicle (1% DMSO) for 30 min, ibrutinib (500 nM) or vehicle (1% DMSO) for 30 min, and finally LPS (100 ng/ml) or PBS for 23 h. Consistent with our findings above, ibrutinib significantly suppressed LPS-induced BV2 microglial cell migration compared with LPS treatment (Fig. 8a, b). More importantly, treatment with MK2206, ibrutinib, and LPS did not alter LPS-induced BV2 microglial cell migration compared with treatment with MK2206 and LPS or ibrutinib and LPS (Fig. 8a, b), suggesting that ibrutinib alters LPS-stimulated BV2 microglial cell migration via AKT signaling.

Next, we tested whether ibrutinib modulates LPS-induced BV2 microglial cell migration in a STAT3-dependent manner (Fig. 8c, d). BV2 microglial cells were scratched using a fine pipette tip and immediately imaged (0 h), followed by treatment with S31-201 (a STAT3 inhibitor, 25 μM) or vehicle (1% DMSO) for 30 min, ibrutinib (500 nM) or vehicle (1% DMSO) for 30 min, and finally LPS (100 ng/ml) or PBS for 23 h. Again, ibrutinib significantly reduced LPS-induced BV2 microglial cell migration (Fig. 8c, d). In addition, treatment with S31-201, ibrutinib, and LPS further decreased LPS-induced BV2 microglial cell migration compared with treatment with S31-201 and LPS (Fig. 8c, d). These data suggest that the modulation of LPS-induced BV2 microglial cell migration by ibrutinib does not require STAT3 signaling.

### Ibrutinib inhibits LPS-stimulated microglial and astrocyte activation in wild-type mice

**Fig. 9:**
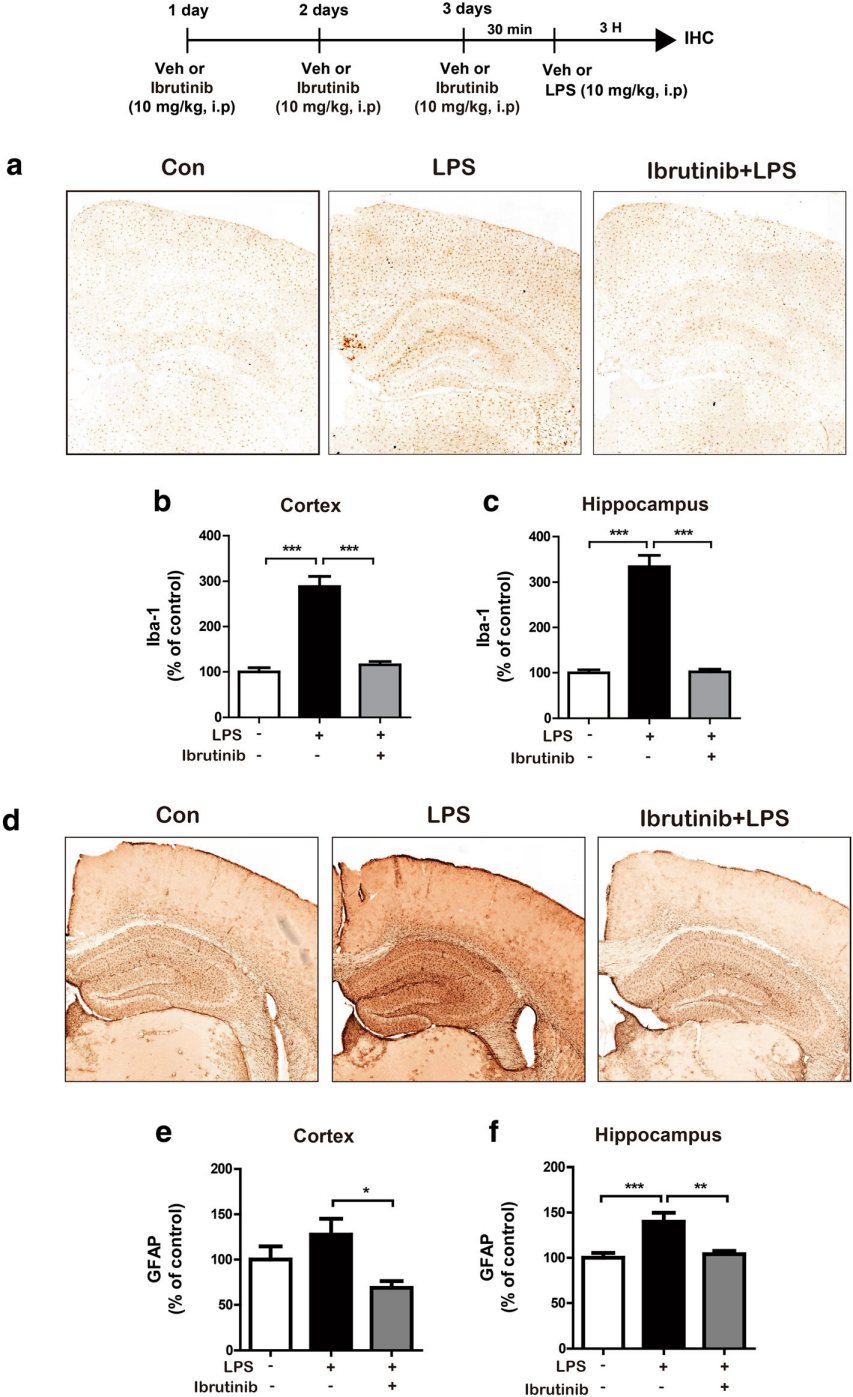
Ibrutinib significantly reduced LPS-stimulated microglial and astrocyte activation in wild-type mice. **a** Wild-type mice were injected with ibrutinib (10 mg/kg, i.p.) or vehicle (10% DMSO, i.p.) daily for 3 days, followed by injection with LPS (10 mg/kg, i.p.) or PBS. Three hours after the injection of LPS or PBS, immunohistochemistry was conducted with an anti-Iba-1 antibody. **b**, **c** Quantification of the data shown in **a** (cortex: vehicle, *n* = 5 mice; LPS, *n* = 5 mice; ibrutinib+LPS, *n* = 5 mice; and hippocampus: vehicle, *n* = 5 mice; LPS, *n* = 5 mice; ibrutinib+LPS, *n* = 5 mice). **d** Wild-type mice were injected with ibrutinib (10 mg/kg, i.p.) or vehicle (10% DMSO, i.p.) daily for 3 days, followed by injection with LPS (10 mg/kg, i.p.) or PBS. Three hours after the injection of LPS or PBS, immunohistochemistry was conducted with an anti-GFAP antibody. **e**, **f** Quantification of the data shown in **d** (cortex: con, *n* = 5 mice; LPS, *n* = 5 mice; ibrutinib+LPS, *n* = 5 mice; and hippocampus: con, *n* = 5 mice; LPS, *n* = 5 mice; ibrutinib+LPS, *n* = 5 mice). *p < 0.05, **p < 0.01, ***p < 0.001

According to recent studies, activated microglia and astrocytes are associated with proinflammatory responses and neuroinflammation [7]. Thus, we examined the effects of ibrutinib on LPS-induced microglial and astrocyte activation in vivo. Wild-type mice were first injected with ibrutinib (10 mg/kg, i.p.) daily for 3 days and then injected with LPS (10 mg/kg, i.p.) or PBS. Three hours after injection with LPS or PBS, immunohistochemistry was conducted with anti-Iba-1 or anti-GFAP antibodies. The LPS-injected wild-type mice showed a significant increase in microglial and astrocyte activation (Fig. 9), whereas ibrutinib significantly inhibited microglial (Fig. 9a–c) and astrocyte (Fig. 9d–f) activation in the cortex and hippocampus of LPS-injected wild-type mice.

**Fig. 10:**
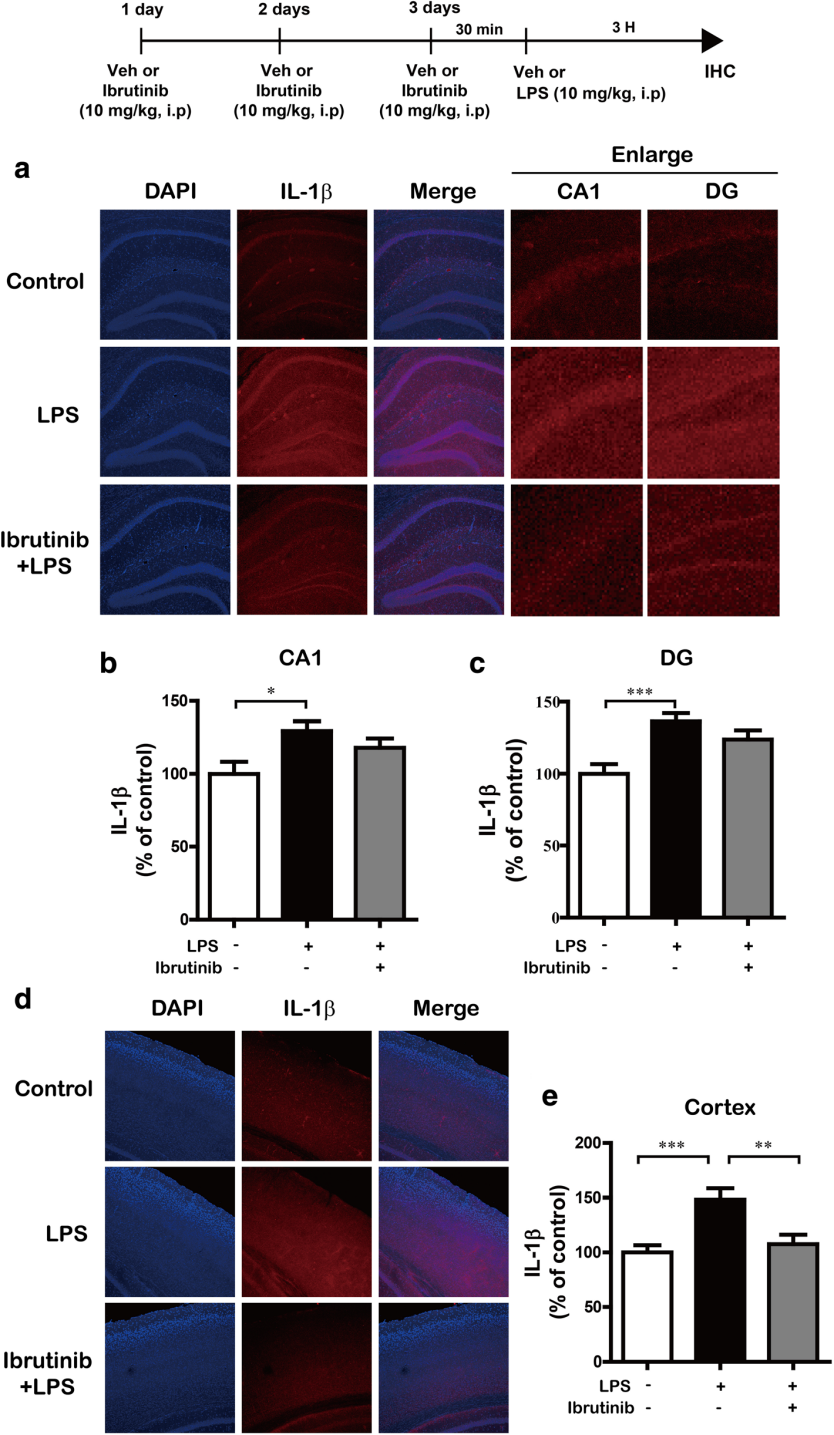
Ibrutinib significantly decreased LPS-induced IL-1β levels in vivo. **a** Wild-type mice were injected with ibrutinib (10 mg/kg, i.p.) or vehicle (10% DMSO, i.p.) daily for 3 days, followed by injection with LPS (10 mg/kg, i.p.) or PBS. Three hours after the injection of LPS or PBS, the mice were perfused, fixed, and immunostained with an anti-IL-1β antibody in the hippocampus. **b**, **c** Quantification of the data from **a** (hippocampus: con, *n* = 5 mice; LPS, *n* = 5 mice; ibrutinib+LPS, *n* = 5 mice). **d** Wild-type mice were injected with ibrutinib (10 mg/kg, i.p.) or vehicle (10% DMSO, i.p.) daily for 3 days, followed by injection with LPS (10 mg/kg, i.p.) or PBS. Three hours after the injection of LPS or PBS, the mice were perfused, fixed, and immunostained with an anti-IL-1β antibody in the cortex. **e** Quantification of the data from **d** (cortex: con, *n* = 5 mice; LPS, *n* = 5 mice; ibrutinib+LPS, *n* = 5 mice). *p < 0.05, **p < 0.01, ***p < 0.001

**Fig. 11:**
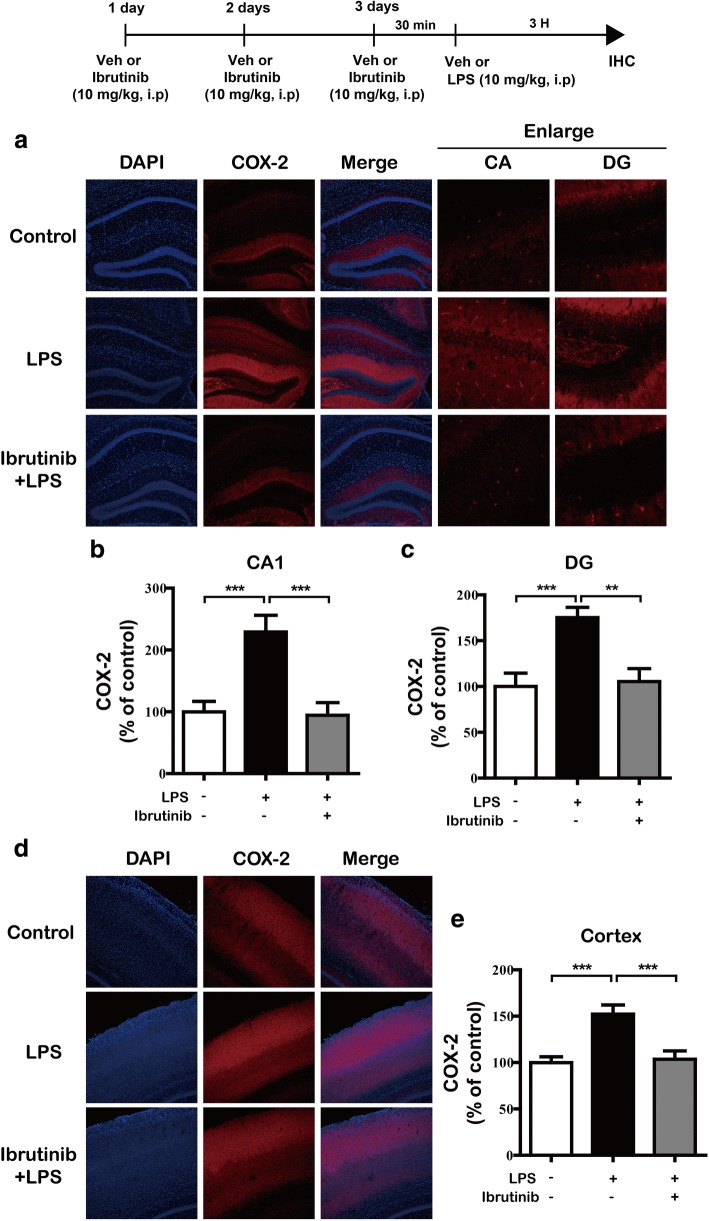
Ibrutinib significantly downregulated LPS-stimulated COX-2 levels in vivo. **a** Wild-type mice were injected with ibrutinib (10 mg/kg, i.p.) or vehicle (10% DMSO, i.p.) daily for 3 days, followed by injection of LPS (10 mg/kg, i.p.) or PBS. Three hours after the injection of LPS or PBS, the mice were perfused, fixed, and immunostained with an anti-COX-2 antibody in the hippocampus. **b**, **c** Quantification of the data from **a** (hippocampus: con, *n* = 4 mice; LPS, *n* = 4 mice; ibrutinib+LPS, *n* = 4 mice). **d** Wild-type mice were injected with ibrutinib (10 mg/kg, i.p.) or vehicle (10% DMSO, i.p.) daily for 3 days, followed by injection with LPS (10 mg/kg, i.p.) or PBS. Three hours after the injection of LPS or PBS, the mice were perfused, fixed, and immunostained with an anti-COX-2 antibody in the cortex. **e** Quantification of the data from **d** (cortex: con, *n* = 4 mice; LPS, *n* = 4 mice; ibrutinib+LPS, *n* = 4 mice). **p < 0.01, ***p < 0.001

Next, we investigated whether ibrutinib regulates the LPS-stimulated increase in IL-1β and COX-2 levels in LPS-injected wild-type mice. For this experiment, wild-type mice were first injected with ibrutinib (10 mg/kg, i.p.) daily for 3 days and then injected with LPS (10 mg/kg, i.p.) or PBS. Three hours after the injection with LPS or PBS, immunohistochemistry was conducted with anti-IL-1β or anti-COX-2 antibodies. The LPS-injected wild-type mice exhibited a significant increase in IL-1β levels compared with vehicle-injected wild-type mice (Fig. 10a–e). In addition, ibrutinib were significantly decreased IL-1β levels in the cortex but not the hippocampus in LPS-injected wild-type mice (Fig. 10). Moreover, ibrutinib were significantly downregulated COX-2 levels in the hippocampus (Fig. 11a–c) and cortex (Fig. 11d, e) in LPS-injected wild-type mice. These data suggest that ibrutinib modulates LPS-induced microglial and astrocyte activation as well as the levels of the proinflammatory cytokines COX-2 and IL-1β in vivo.

## Discussion

Microglia and astrocytes are the first line of defense in the central nervous system (CNS) and initiate immune responses to injuries and pathogens [24]. Activated microglia and astrocytes release a variety of proinflammatory cytokines [25, 26]. Specifically, abnormally activated microglia produce a variety of inflammatory mediators (COX-2 and iNOS) and inflammatory cytokines (IL-1β, IL-6, and TNF-α). During pathological conditions involving CNS inflammation, IL-1β is mainly released by activated macrophages and microglia, and astrocytes are regarded as the major target of IL-1β, as suggested by the presence of IL-1β receptors on the surfaces of astrocytes [27]. In astrocytes, IL-1β induces the expression of other cytokines, including IL-6 and TNF-α, as well as other inflammatory mediators that have been implicated in the CNS immune response to injury [28]. Interestingly, systemic LPS and IL-1β injections have been reported to induce excess COX-2 production within the rodent brain [29, 30]. The COX-2 expression is substantially increased in the frontal cortex and hippocampus in the brains of subjects with Alzheimer’s disease (AD) [31]. Therefore, drugs modulating microglial activation and the release of proinflammatory cytokines that effectively inhibit inflammation represent a promising therapeutic strategy for neuroinflammation/neurodegeneration-related diseases. Not surprisingly, ibrutinib itself did not alter proinflammatory cytokine levels in BV2 microglial cells compared with vehicle treatment in the present study (Fig. 2, Additional file 1: Figure S1), suggesting that ibrutinib alone does not affect the levels of any proinflammatory cytokines under basal conditions. However, ibrutinib significantly reduced proinflammatory cytokine levels in LPS-induced BV2 microglia (Fig. 2, Additional file 1: Figure S1) and primary microglia (Fig. 3) but not primary astrocytes (Additional file 1: Figure S2). In addition, pretreatment with ibrutinib reduced proinflammatory cytokine levels more effectively than post-treatment, highlighting the potential of ibrutinib as a preventive drug (Fig. 2, Additional file 1: Figure S1). Based on these findings, we speculate that pre- or post-treatment with ibrutinib differentially modulates LPS-induced proinflammatory cytokine production depending on the cell type.

The members of the TLR family are the main mediators of the innate immune response. TLRs are mainly expressed in immune cells and have also been identified in different CNS cell types, such as microglia, astrocytes, or cells in the cerebral microvasculature [32]. TLR4 is the most representative member of the TLR family and predominantly responds to LPS through its co-receptor, myeloid differentiation protein-2 (MD-2), which is essential for LPS-induced stimulation of TLR4 [33]. TLR4 binds to some other adapter proteins, including myeloid differentiation factor 88 (MyD88), to activate downstream signaling. Specifically, the interaction between LPS and TLR4 activates MARK signaling pathways (including AKT) in BV2 microglial cells [34]. Therefore, abnormal TLR4 expression or abnormal immune responses might damage the CNS. Interestingly, in the present study, treatment with TAK-242 (a TLR4 inhibitor), ibrutinib, and LPS further decreased LPS-induced COX-2 mRNA levels compared with the treatment with TAK-242 and LPS or ibrutinib and LPS (Fig. 4a, b). However, treatment with TAK-242, ibrutinib, and LPS did not reduce LPS-induced IL-1β mRNA levels compared with treatment with TAK-242 and LPS or ibrutinib and LPS (Fig. 4a–c). These data suggest that ibrutinib alters TLR4 and/or other neuroinflammation-related receptors to modulate LPS-induced proinflammatory cytokine levels.

How does ibrutinib downregulate proinflammatory cytokine levels? Ibrutinib may inhibit the interaction between LPS and TLR4 on the cell surface and thereby deactivate downstream signaling pathways to suppress the neuroinflammatory response. Interestingly, we found that ibrutinib decreased LPS-induced cell-surface levels of TLR4 compared with LPS treatment (Additional file 1: Figure S3a, b). Another possible mechanism is that ibrutinib directly or indirectly suppresses TLR4 activation to reduce neuroinflammatory responses via other neuroinflammatory-related receptors that interact with LPS. Based on our findings, ibrutinib may regulate cell-surface levels of TLR4 to inhibit the interaction between TLR4 and LPS on the cell surface to alter neuroinflammatory responses. Future studies will examine whether ibrutinib modulates the LPS and TLR4 interaction and/or other neuroinflammatory-related receptors to regulate neuroinflammation.

AKT signaling plays an important role in the LPS-induced proinflammatory response [35]. AKT is the main kinase in the signal transduction pathway predominantly responsible for the production and synthesis of proinflammatory mediators and modulates TLR4 expression [36]. For instance, AKT negatively regulates LPS-induced TNF-α and IL-6 levels in the bone marrow macrophages [37]. Phosphorylated AKT also promotes the expression of inflammatory molecules, including iNOS and COX-2 [38]. As shown by Saponaro et al., LPS binds to TLR4 and activates AKT signaling to alter the production of the proinflammatory cytokine iNOS in microglial cells [39]. Thus, the maintenance of a homeostatic balance in AKT signaling might play an important role in its anti-inflammatory effects. In the present study, we found that ibrutinib dramatically reduced LPS-induced AKT phosphorylation in BV2 microglial cells (Fig. 5). Unexpectedly, we observed that ibrutinib further decreased LPS-induced AKT phosphorylation compared with vehicle to below basal levels (ibrutinib+LPS vs vehicle, Fig. 5). We therefore tested whether ibrutinib itself alters p-AKT levels and found that ibrutinib alone significantly reduced p-AKT levels compared with vehicle treatment (Additional file 1: Figure S5). Since ibrutinib alone decreased p-AKT levels, we investigated whether ibrutinib itself regulates proinflammatory cytokine levels and found that ibrutinib alone did not reduce any proinflammatory cytokine levels compared with vehicle treatment (Fig. 2, Additional file 1: Figure S1). However, AKT inhibition selectively regulated LPS-induced proinflammatory cytokine levels in the presence of ibrutinib (Fig. 5g–i). Based on our findings and the literature, we suggest that ibrutinib inhibits AKT phosphorylation to alter LPS-induced neuroinflammatory responses. In addition, ibrutinib itself may affect anti-inflammatory cytokine levels to regulate neuroinflammatory responses or may affect another biological function (i.e., phagocytosis) in the presence/absence of LPS in BV2 microglial cells. Future studies will explore whether ibrutinib itself modulates anti-inflammatory effects and how ibrutinib regulates p-AKT levels in the absence of LPS, as well as the molecular mechanisms by which ibrutinib differentially regulates neuroinflammatory responses and/or other biological functions in the absence/presence of LPS in microglial cells.

STAT3 is a transcription factor that plays a critical role in neuroinflammatory responses [40, 41]. STAT3 homodimerizes, autophosphorylates, translocates to the nucleus, and binds to the enhancers in the IL-6 promoter to induce gene transcription [42]. STAT3 expression was recently shown to be upregulated in BV2 microglial cells in response to LPS [43]. In addition, levels of the proinflammatory cytokines IL-6 and IL-10 are highly dependent on STAT3 signaling [44]. Activated STAT3 also regulates the levels of other inflammatory cytokines to promote immune responses. Here, ibrutinib alone can decrease p-STAT3 levels compared with vehicle treatment (Additional file 1: Figure S5). In addition, ibrutinib significantly suppressed LPS-induced STAT3 signaling and nuclear p-STAT3 (Ser727) levels in BV2 microglial cells (Fig. 6). Moreover, several studies have shown that LPS-induced STAT3 activation results in increased iNOS expression via the mTOR or MAPK pathway in murine macrophage-like cells [45, 46]. Additionally, Murase and McKay et al. reported that inhibition of AKT with LY294002 blocks STAT3 phosphorylation at Ser727, suggesting that the AKT pathway is responsible for STAT3-Ser727 phosphorylation in rat hippocampal neurons [47]. Based on the literature and our findings, we hypothesized that ibrutinib affects AKT and/or STAT3 signaling and modulates LPS-induced nuclear p-STAT3 (Ser 727) levels to alter neuroinflammatory responses. However, ibrutinib altered LPS-induced nuclear p-STAT3 (Ser 727) levels in an AKT-independent manner in our systems (Additional file 1: Figure S6). Thus, there are several possible routes by which ibrutinib might affect AKT and/or STAT3 signaling to influence neuroinflammation. One possibility is that ibrutinib affects LPS-induced AKT signaling to alter another potential LPS-induced transcription factor (e.g., p-NF-κB) in the nucleus to alter neuroinflammatory responses. A second possibility is that ibrutinib regulates LPS-induced STAT3 signaling in the cytosol, thereby modulating LPS-induced nuclear p-STAT3 (Ser 727) levels as a downstream target and leading to altered neuroinflammation in BV2 microglial cells. It is also possible that ibrutinib influences other neuroinflammation-related signaling pathways (e.g., mTOR signaling) to affect known/unknown transcription factors and thus alter LPS-induced proinflammatory cytokine production. Future studies will determine whether ibrutinib regulates other neuroinflammation-related signaling pathways and/or unknown transcription factors to modulate the levels of individual proinflammatory cytokines.

Microglial cell migration is associated with stimulation of microglial cells, which causes chronic inflammation and neuronal damage [48]. For example, chemokines released from microglial cells are key components required for cell movement. LPS-induced migration of BV2 microglial cells requires the activation of the AKT signaling pathway [49]. In addition, candidate compounds with anti-inflammatory effects strongly inhibit LPS-induced BV2 cell migration by inhibiting NF-κB/STAT3 [23, 50]. In the present study, ibrutinib significantly suppressed LPS-mediated BV2 microglial cell migration (Fig. 7). Thus, we hypothesized that ibrutinib may modulate microglial cell migration by altering the LPS-stimulated increases in the levels of proinflammatory cytokines and/or AKT/STAT3 signaling. To test our hypothesis, we first determined whether ibrutinib itself alters BV2 microglial cell migration because we observed that ibrutinib alone downregulated p-AKT signaling compared with vehicle treatment in the absence of LPS (Additional file 1: Figure S5). However, we observed that ibrutinib alone did not reduce BV2 microglial cell migration compared with vehicle (Additional file 1: Figure S7), suggesting that ibrutinib itself may affect other unknown functions (e.g., phagocytosis) in the absence of LPS in BV2 microglial cells. We then conducted wound-healing assays in which LPS-induced BV2 microglial cells were treated with an AKT inhibitor and ibrutinib or a STAT3 inhibitor and ibrutinib and found that ibrutinib altered LPS-induced BV2 microglial cell migration via AKT signaling but not STAT3 signaling (Fig. 8). Based on our findings, we speculate that ibrutinib differentially affects AKT and STAT3 signaling to selectively regulate proinflammatory cytokine levels and/or microglial cell migration. Further studies are required to fully dissect the multiple molecular mechanisms involved in ibrutinib-mediated microglial cell migration.

Systemic injections of LPS promote microglial and astrocyte activation and increase proinflammatory cytokine levels in wild-type mice [51]. Skelly et al. found that even a single injection of LPS induces a robust expression of the proinflammatory cytokines IL-1β and COX-2 in the hippocampus in wild-type mice [52]. LPS induces neuroinflammation in the mouse brain, as evidenced by increased immunostaining for Iba-1 (for microglial cells) and GFAP (for astrocytes) [53]. In the present study, LPS-injected wild-type mice exhibited significantly increased microglial and astrocyte activation. Ibrutinib strongly inhibited this LPS-mediated microglial and astrocyte activation (Fig. 9) as well as the increase in COX-2 and IL-1β proinflammatory cytokine levels (Figs. 10 and 11), suggesting that ibrutinib has potential as a targeted drug for neuroinflammation-related diseases.

## Conclusions

In summary, ibrutinib significantly reduced the LPS-mediated increases in proinflammatory cytokine levels in BV2 microglial and primary microglial cells. In addition, ibrutinib alter TLR4 to regulate LPS-induced increases in proinflammatory cytokine levels in BV2 microglial cells. Ibrutinib significantly decreased LPS-stimulated downstream AKT and STAT3 signaling to alter the neuroinflammatory response and/or microglial cell migration. Moreover, ibrutinib-treated wild-type mice exhibited significantly reduced microglial/astrocyte activation and proinflammatory cytokine levels. Thus, we suggest that ibrutinib may a potential therapeutic agent for neuroinflammation-related diseases.

## Additional file

Additional file 1: **Figure S1.** Post-treatment with ibrutinib significantly decreased LPS-induced pro-inflammatory cytokine COX-2, IL-6, and iNOS mRNA levels. **Figure S2.** Ibrutinib did not reduce any LPS-mediated increases in pro-inflammatory cytokine levels in primary astrocytes. **Figure S3.** Ibrutinib decreased LPS-induced cell-surface levels of TLR4s. **Figure S4.** Ibrutinib did not reduce LPS-induced ERK/JNK/P38 signaling. **Figure S5.** Ibrutinib itself decreased p-AKT and p-STAT3 levels compared to vehicle treatment. **Figure S6.** Ibrutinib modulates LPS-induced nuclear p-STAT3 (Ser 727) levels in an AKT-independent manner. **Figure S7.** Ibrutinib itself did not reduce BV2 microglial cell migration. (DOCX 17300 kb)

## Abbreviations

AD: Alzheimer’s disease
BTK: Bruton’s tyrosine kinase
CLL: Chronic lymphocytic leukemia
COX-2: Cyclooxygenase-2
IL-1β: Interleukin-1β
IL-6: Interleukin-6
LPS: Lipopolysaccharide
STAT3: Signal transducer and activator of transcription 3
TLR4: Toll-like receptor 4
TNF-α: Tumor necrosis factor-α
