# Supplementary material for: Ibrutinib suppresses LPS-induced neuroinflammatory responses in BV2 microglial cells and wild-type mice
Source: J Neuroinflammation. 2018 Sep 19;15:271. doi: 10.1186/s12974-018-1308-0 (PMC6145206; doi:10.1186/s12974-018-1308-0)
Supplement: Supplementary file 1 — Figure S1. Post-treatment with ibrutinib significantly decreased LPS-induced pro-inflammatory cytokine COX-2, IL-6, and iNOS mRNA levels. Figure S2. Ibrutinib did not reduce any LPS-mediated increases in pro-inflammatory cytokine levels in primary astrocytes. Figure S3. Ibrutinib decreased LPS-induced cell-surface levels of TLR4s. Figure S4. Ibrutinib did not reduce LPS-induced ERK/JNK/P38 signaling. Figure S5. Ibrutinib itself decreased p-AKT and p-STAT3 levels compared to vehicle treatment. Figure S6. Ibrutinib modulates LPS-induced nuclear p-STAT3 (Ser 727) levels in an AKT-independent manner. Figure S7. Ibrutinib itself did not reduce BV2 microglial cell migration. (DOCX 17300 kb) [file 12974_2018_1308_MOESM1_ESM.docx]

**Ibrutinib suppresses LPS-induced neuroinflamatory responses in BV2 microglial cells and wild-type mice.**

Hye Yeon Nam^1,2^, Jin Han Nam^1,2^, Gwangho Yoon^1,2^, Ju-Young Lee^1,2^, Youngpyo Nam^1,2^, Hye-Jin Kang^1^, Hyun-Ji Cho^1^, Jeongyeon Kim^1^, Hyang-Sook Hoe^1,*^

^1^Department of Neural Development and Disease, Korea Brain Research Institute (KBRI), 61, Cheomdan-ro, Dong-gu, Daegu, Korea 41068

^2^These authors contributed equally to this work.

Hye Yeon Nam: hynam@kbri.re.kr

Jin Han Nam:jinhan02@naver.com

Gwangho Yoon: [ghyoon@kbri.re.kr](mailto:ghyoon@kbri.re.kr)

Ju-Young Lee:zf79@kbri.re.kr

Youngpyo Nam: ypnam@kbri.re.kr

Hye-Jin Kang:khyejin1057@naver.com

Hyun-Ji Cho: [hjcho.dr@gmail.com](mailto:hjcho.dr@gmail.com)

Jeongyeon Kim: jykim@kbri.re.kr

Hyang-Sook Hoe: [sookhoe72@kbri.re.kr](mailto:sookhoe72@kbri.re.kr)

^*^Corresponding author

Hyang-Sook Hoe, Ph.D., Department of Neural Development and Disease, Korea Brain Research Institute (KBRI), 61, Cheomdan-ro, Dong-gu, Daegu, Korea 41068

E-mail: [sookhoe72@kbri.re.kr](mailto:sookhoe72@kbri.re.kr)

**
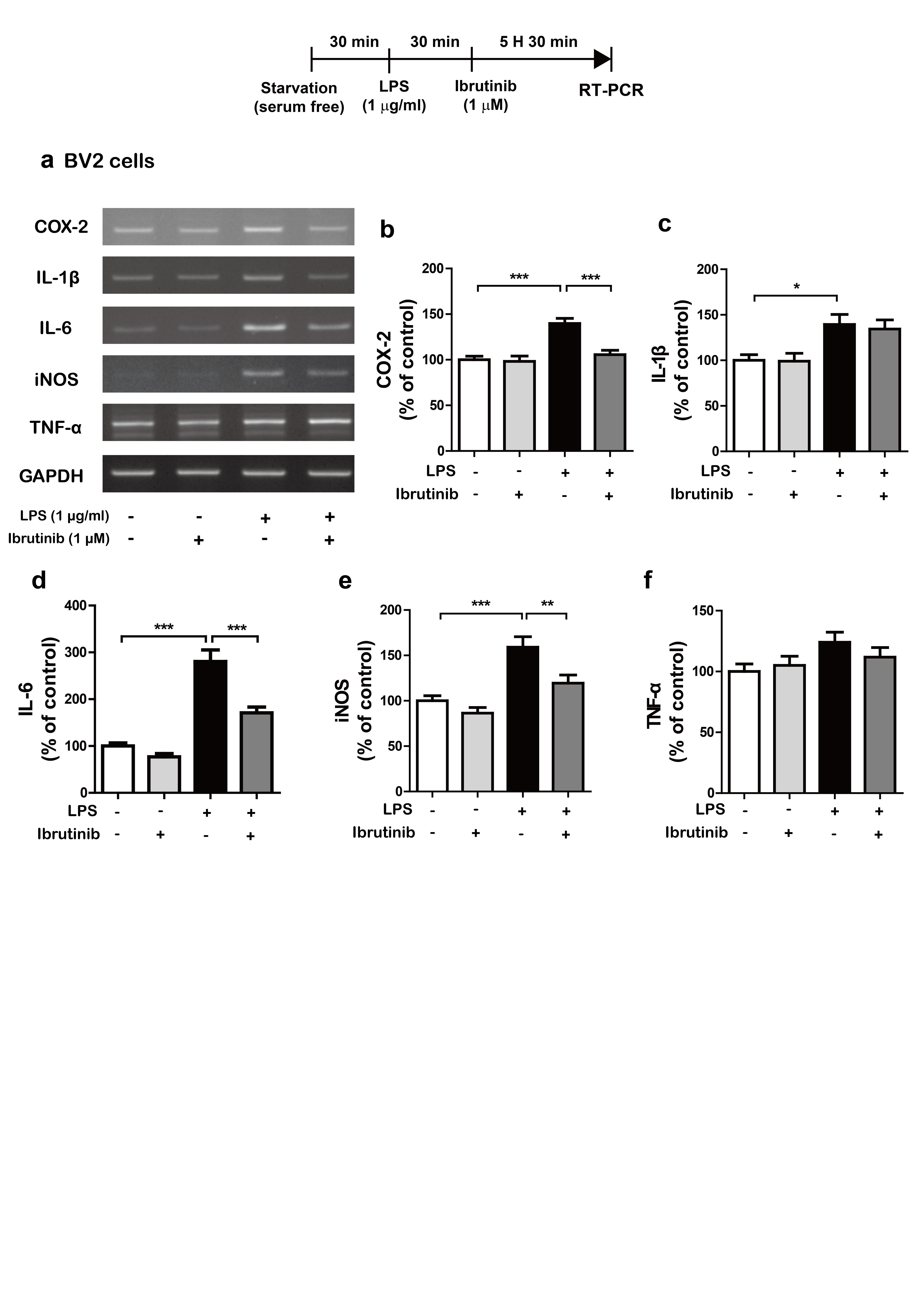
**

**Figure S1** Post-treatment with ibrutinib significantly decreased LPS-induced pro-inflammatory cytokine COX-2, IL-6, and iNOS mRNA levels. **a** BV2 microglial cells were pre-treated with LPS (1 μg/ml) or PBS for 30 min, treated with ibrutinib (1 μM) or vehicle (1% DMSO) for 5.5 hr, and pro-inflammatory cytokine levels were measured using RT-PCR. **b-f** Quantification of data from **a** (COX-2, IL-1β, IL-6, iNOS, and TNF-α : con, n=17; Ibrutinib, n=17, LPS, n=17; Ibrutinib+LPS, n=17). *p<0.05, **p<0.01, ***p<0.001.


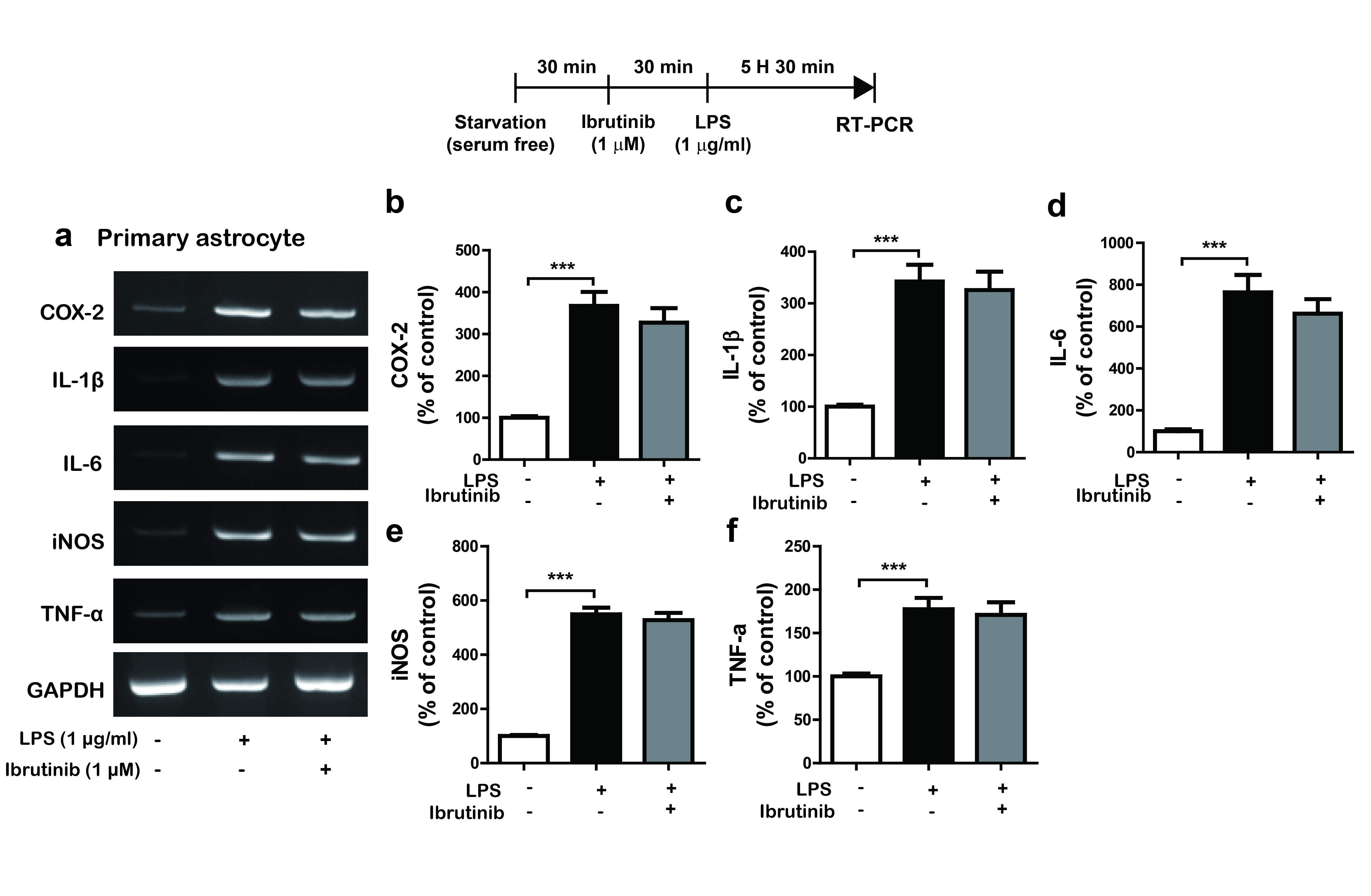


**Figure S2.** Ibrutinib did not reduce any LPS-mediated increases in pro-inflammatory cytokine levels in primary astrocytes. **a** Primary astrocytes were treated with vehicle (1% DMSO) or Ibrutinib (1 μM) for 30 min, treated with PBS or LPS (1 μg/ml) for 5 hr 30 min, and RT-PCR were conducted. **b-f** Quantification of data from **a** (COX-2, IL-1β, IL-6 and iNOS: con, n=16; LPS, n=16; Ibrutinib+LPS, n=16; TNF-α: con, n=8; LPS, n=8; Ibrutinib+LPS, n=8). ***p<0.001.

**
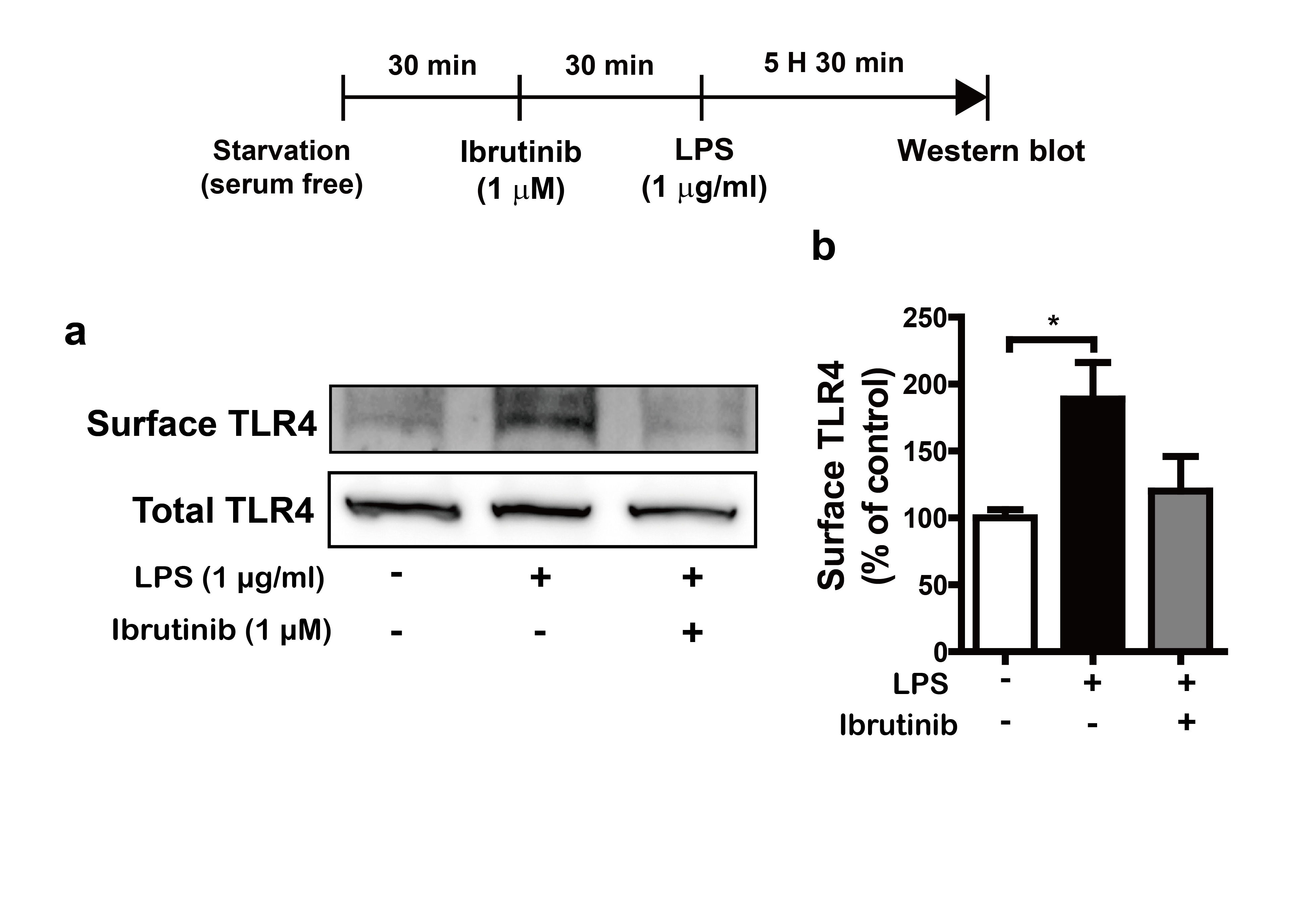
**

**Figure S3** Ibrutinib decreased LPS-induced cell-surface levels of TLR4s. **a** BV2 microglial cells were pretreated with ibrutinib (1 μM) or vehicle (1% DMSO) for 30 min, followed by treatment with LPS (1 μg/ml) or PBS for 5.5 hr, and cell surface biotinylation was performed to measure cell-surface levels of TLR4. To measure the total levels of TLR4, BV2 microglial cells were pretreated with ibrutinib (1 μM) or vehicle (1% DMSO) for 30 min, followed by treatment with LPS (1 μg/ml) or PBS for 5.5 hr, and western blotting was conducted to measure the total levels of TLR4. **b** Quantification of the data from **a** (surface TLR4: con, n=5; LPS, n=5; ibrutinib+LPS, n=5, LPS vs ibrutinib+LPS, p=0.1, by 68.7%). *p<0.05.

**
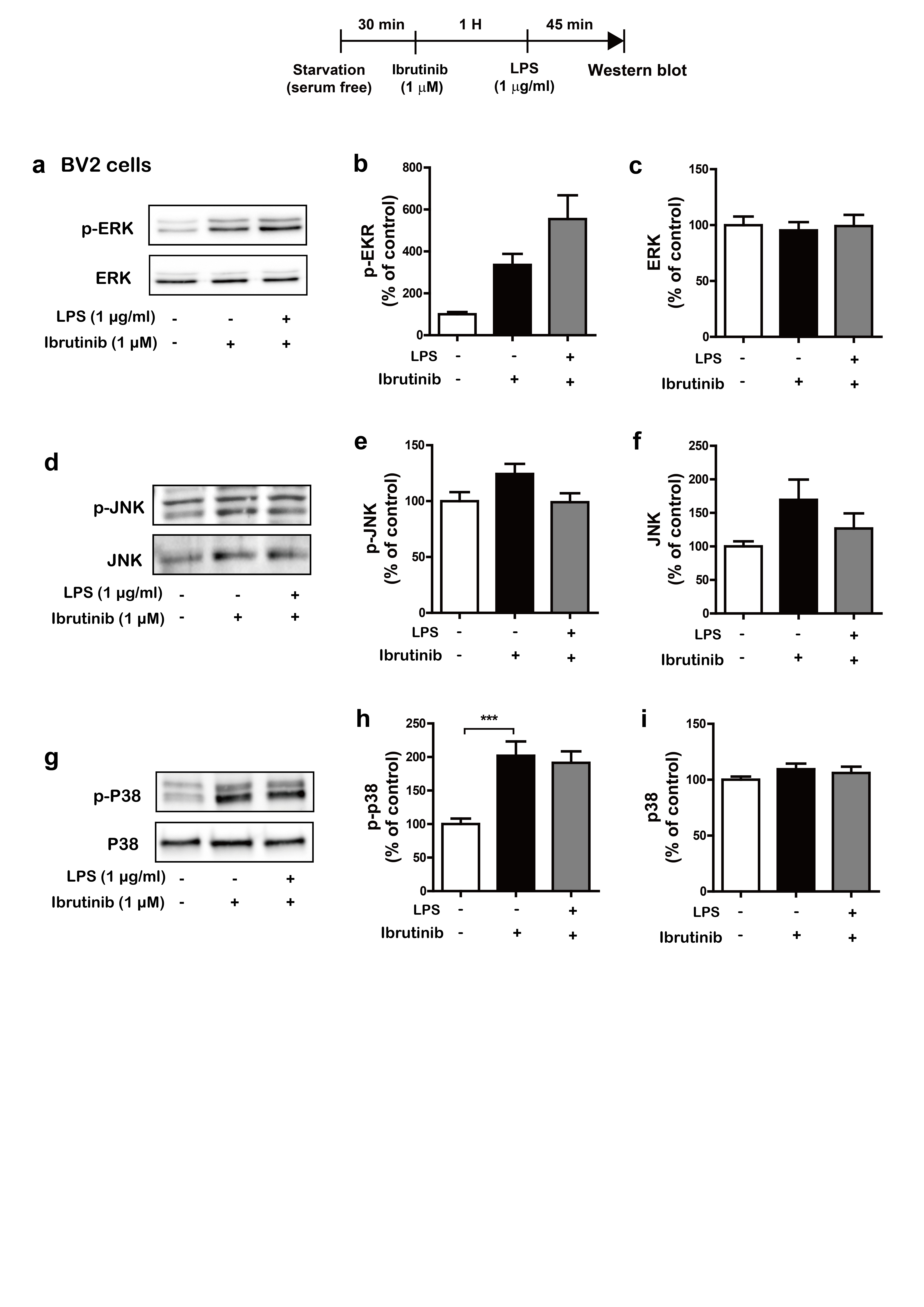
**

**Figure S4** Ibrutinib did not reduce LPS-induced ERK/JNK/P38 signaling. **a, d, g** BV2 microglial cells were treated with vehicle (1% DMSO) or ibrutinib (1 μM) for 1 hr, treated with PBS or LPS (1 μg/ml) for 45 min, and western blotting were performed with anti-p-ERK/ERK, anti-p-JNK/JNK, or anti-p-P38/P38 antibodies. **b-c** Quantification of data from **a** (p-ERK and ERK: con, n=12; LPS, n=12; ibrutinib+LPS, n=12). **e-f** Quantification of data from **d** (p-JNK :con, n=22; LPS, n=22; Ibrutinib+LPS, n=22 and JNK: con, n=12; LPS, n=12; Ibrutinib+LPS, n=12). **h-i** Quantificaiton of data from **g** (p-P38: con, n=15; LPS, n=15; Ibrutinib+LPS, n=15 and P38: con, n=12; LPS, n=12; Ibrutinib+LPS, n=12). ***p<0.001.


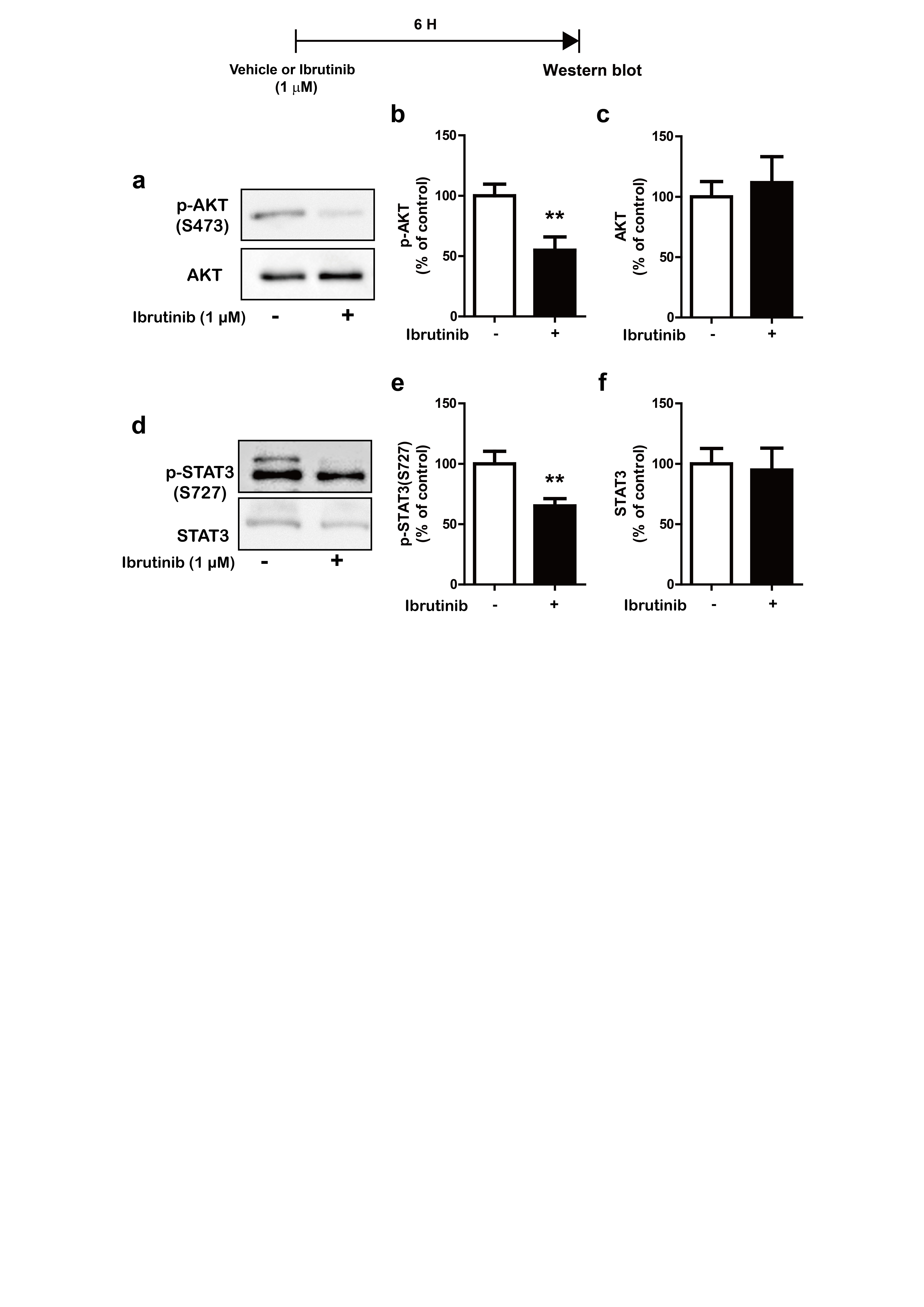


**Figure S5** Ibrutinib itself decreased p-AKT and p-STAT3 levels compared to vehicle treatment. **a** BV2 microglial cells were treated with vehicle (1% DMSO) or ibrutinib (1 μM) for 6 hr and western blotting were performed with anti-p-AKT and anti-AKT antibodies. **b-c** Quantification of data from **a** (p-AKT: con, n=14; Ibrutinib, n=14 and AKT: con, n=14; Ibrutinib, n=14). **d** BV2 microglial cells were treated with vehicle (1% DMSO) or Ibrutinib (1 μM) for 6 hr and western blotting were performed with anti-p-STAT3 and anti-STAT3 antibodies. **e-f** Quantification of data from **d** (p-STAT3: con, n=14; Ibrutinib, n=14 and STAT3: con, n=14; Ibrutinib+LPS, n=14). **p<0.01.





**Figure S6** Ibrutinib modulate LPS-induced nuclear p-STAT3 (Ser 727) levels in an AKT-independent manner. **a** BV2 microglial cells were pretreated with MK2206 (an AKT inhibitor, 10 μM) or vehicle (1% DMSO) for 30 min, treated with ibrutinib (1 μM) or vehicle (1% DMSO) for 30 min, and treated with LPS (1 μg/ml) or PBS for 5 hr. The immunocytochemistry were performed anti-CD11b and anti-p-STAT3 antibodies. **b** Quantification of data from **a (**con, n=1270/cells; LPS, n=1626/cells; ibrutinib+LPS, n=1184/cells; MK2206+LPS, n=800/cells; MK2206+ibrutinib+LPS, n=841/cells**).** *p<0.05, ***p<0.001.


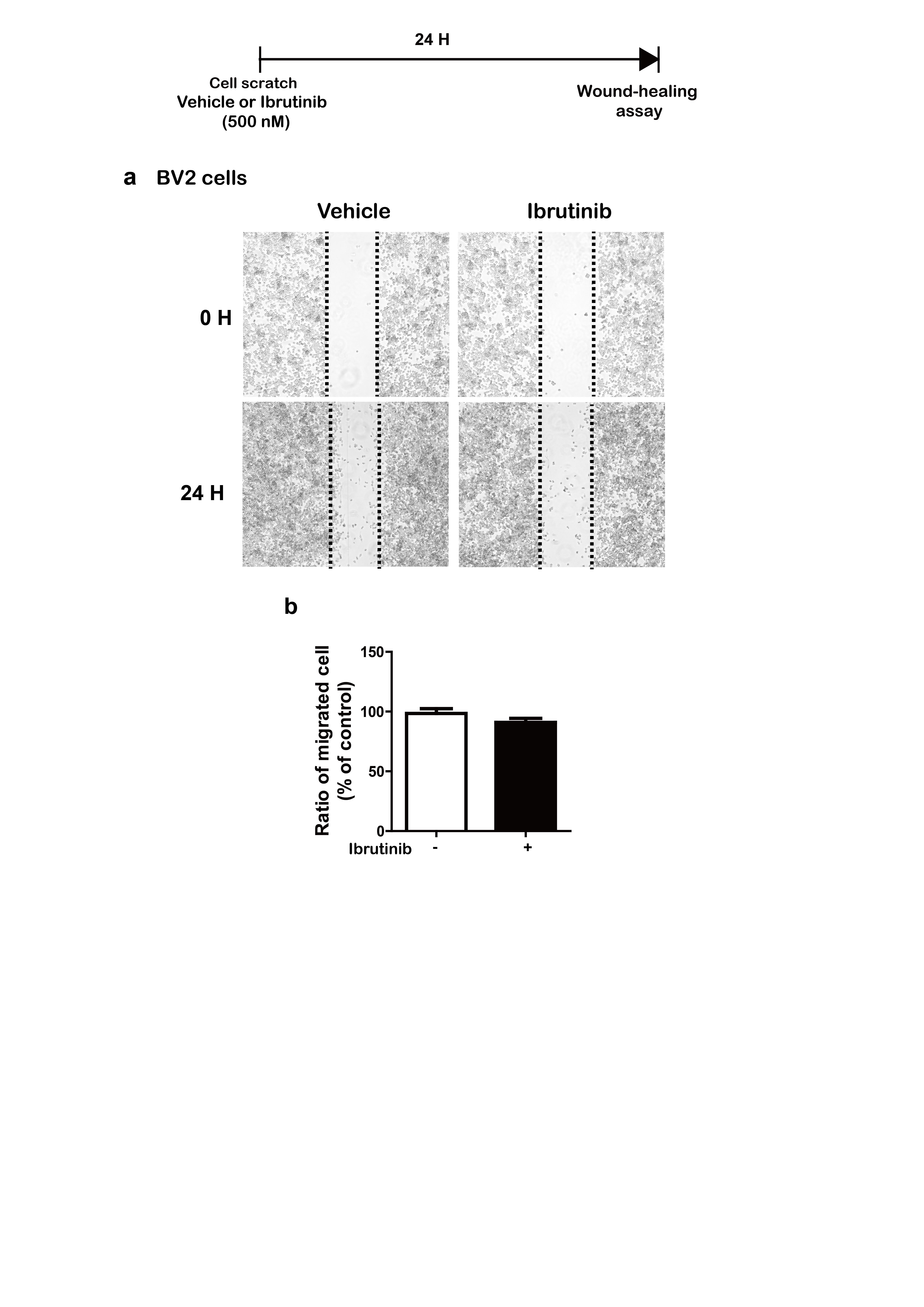


**Figure S7** Ibrutinib itself did not reduce BV2 microglial cell migration. **a** BV2 microglial cell monolayers were scratched with a fine tip, treated with ibrutinib (500 nM) or vehicle (1% DMSO) for 24 hr. Images of the wound gap were acquired at 0 hr (i.e., immediately after scratching) and after 24 hr. **b** Quantification of data from **a (**vehicle, n=27; Ibrutinib, n=27**).**
